# The Role of Cholesterol Metabolism and Its Regulation in Tumor Development

**DOI:** 10.1002/cam4.70783

**Published:** 2025-03-27

**Authors:** Yongmei Wu, Wenqian Song, Min Su, Jing He, Rong Hu, Youbo Zhao

**Affiliations:** ^1^ Department of Human Histology and Embryology Guizhou Medical University Guiyang Guizhou China; ^2^ Center for Tissue Engineering and Stem Cell Research, Key Laboratory of Regenerative Medicine in Guizhou Province Guizhou Medical University Guiyang Guizhou China; ^3^ Characteristic Key Laboratory of Translational Medicine Research of Cardiovascular and Cerebrovascular Diseases in Guizhou Province Guizhou Medical University Guiyang Guizhou China

**Keywords:** cancer, cholesterol, immune, metabolism, tumor microenvironment

## Abstract

**Background:**

Within the tumor microenvironment, tumor cells undergo metabolic reprogramming of cholesterol due to intrinsic cellular alterations and changes in the extracellular milieu. Furthermore, cholesterol reprogramming within this microenvironment influences the immune landscape of tumors, facilitating immune evasion and consequently promoting tumorigenesis. These biological changes involve modifications in numerous enzymes associated with cholesterol uptake and synthesis, including NPC1L1, SREBP, HMGCR, SQLE, and PCSK9.

**Review:**

This review systematically summarizes the role of cholesterol metabolism and its associated enzymes in cancer progression, examines the mechanisms through which dysregulation of cholesterol metabolism affects immune cells within the tumor microenvironment, and discusses recent advancements in cancer therapies that target cholesterol metabolism.

**Conclusion:**

Targeting cholesterol metabolism‐related enzymes can inhibit tumor growth, reshape immune landscapes, and rejuvenate antitumor immunity, offering potential therapeutic avenues in cancer treatment.

## 1 Introduction

Cholesterol, a derivative of cyclopentane polyhydrophenanthrene with the chemical formula C_27_H_46_O, is the primary steroid compound in mammals and plays a critical role in essential cellular processes [1]. As a crucial lipid component of mammalian cell membranes, cholesterol helps maintain membrane integrity and fluidity, while also contributing to the formation of membrane microstructures. Moreover, it acts as a key regulator in cellular signaling by directly interacting with the cell membrane and activating specific receptors, including steroids, hydroxycholesterol, and bile acid‐oxygenated metabolites [2].

Cellular cholesterol homeostasis is meticulously regulated through an intricate network that controls its synthesis, absorption, export, conversion, esterification, storage, and systemic distribution [3]. More importantly, cholesterol and its metabolites play pivotal roles in both promoting tumorigenesis and suppressing anti‐tumor immune responses. For instance, the deletion of the gene encoding SREBP cleavage‐activating protein (SCAP) in the liver leads to liver damage, which manifests as nonalcoholic steatohepatitis (NASH) and hepatocellular carcinoma (HCC) [4]. Similarly, the specific deletion of ABCA1 and overexpression of fatty acid synthase (FASN) in the mouse prostate promote the progression of prostatic intraepithelial neoplasia (PIN) into invasive prostate cancer [5]. Metabolism‐focused pharmacological screening has identified statins—HMGCR inhibitors—as promising therapeutic agents for melanoma with suppressed PGC1α expression and resistance to B‐Raf inhibitors such as Vemurafenib [6]. Furthermore, Cao D et al. [7] discovered a novel inhibitor targeting the ketosynthase (KS) domain of FASN, demonstrating potent anti‐tumor activity in xenograft models of non‐small cell lung cancer (NSCLC) and melanoma. Meanwhile, research by Li Y et al. [8] suggests that targeting the FASN/HIF1α/SLC7A11 pathway could restore sorafenib sensitivity in HCC cells. Cholesterol synthesis also plays a crucial role in cancer stem cells, which drive tumor growth, recurrence, and therapy resistance, making it a viable target for therapeutic intervention [9, 10]. Additionally, cholesterol functions as a signaling molecule that regulates morphogenetic pathways, such as Hedgehog signaling [11], and modulates various oncogenic pathways, including Wnt/β‐catenin and EGFR‐STAT3 [12, 13]. Collectively, these findings underscore the critical role of dysregulated cholesterol metabolism in cancer initiation and progression.

Therefore, this review first explores the mechanisms regulating cholesterol uptake, including exogenous absorption and de novo synthesis. Next, it discusses the intricate role of cholesterol metabolism in tumorigenesis and the latest advances in the field. Finally, it examines the feasibility of targeting key cholesterol metabolism proteins—such as LXR, SREBP, HMGCR, FPPS, and SQLE—as potential strategies for clinical anti‐tumor therapy.

## 2 Cholesterol Intake

Cholesterol performs several critical functions: it maintains cell membrane structure [14], facilitates cellular communication [15], enhances immunity, and serves as a precursor for the synthesis of steroids, sex hormones, vitamin D [16], bile salts, and oxysterols [17]. Cholesterol is derived from two primary sources: exogenous, with the majority of cholesterol coming from dietary intake [18, 19], and endogenous, where it is synthesized within the body [20]. An increase in exogenous cholesterol can feedback inhibit endogenous cholesterol synthesis [21]. Two main lipoproteins are involved in cholesterol transport: low‐density lipoprotein (LDL) and high‐density lipoprotein (HDL) [22]. LDL transports cholesterol from the liver to peripheral tissues, while HDL transports cholesterol from peripheral tissues back to the liver, maintaining a dynamic equilibrium [23]. In contrast, disturbances in cholesterol homeostasis are considered to be one of the hallmarks of cancer, and the regulation of cholesterol homeostasis can impact the initiation and progression of cancer [24].

### 2.1 Exogenous Acquisition

Cholesterol primarily exists in the form of esters in animal‐derived foods such as meat, fish, eggs, and dairy products. Niemann‐Pick C1‐Like 1 (NPC1L1) plays a pivotal role in the intestinal absorption of cholesterol. NPC1L1 is localized to the brush‐border membrane of enterocytes and the tubular membranes of hepatocytes [25]. It is a polytopic transmembrane protein with four large luminal domains: the extracellular N‐terminal domain (NTD), the middle lumen domain (MLD), the cysteine‐rich domain (CTD), and a transmembrane domain (TMD) consisting of 13 transmembrane helices (TMs) embedded within the membrane [26]. Among the luminal domains, the NTD has been shown to directly bind cholesterol extracellularly [27], while the MLD is reported to contain binding sites for the cholesterol absorption inhibitor ezetimibe [28]. TMs 3 to 7 form the sterol‐sensing domain (SSD), and the SSD‐intervening loops (LoopTM1–TM2, LoopTM3–TM4, LoopTM5–TM6, and LoopTM7–TM8) along with the C‐terminal cytoplasmic tail are positioned on the cytoplasmic side [29]. The SSD responds to fluctuations in cholesterol levels by binding varying amounts of cholesterol molecules [30]. LoopTM7–TM8 is considered a crucial component of cholesterol uptake [31], and an endocytosis signal peptide (YVNXXF, where X represents any amino acid) has been identified at the cytoplasmic C‐terminal tail of NPC1L1. This peptide binds to Numb, a clathrin adapter protein, and recruits clathrin to mediate the endocytosis of NPC1L1‐cholesterol complexes [32].

**FIGURE 1:**
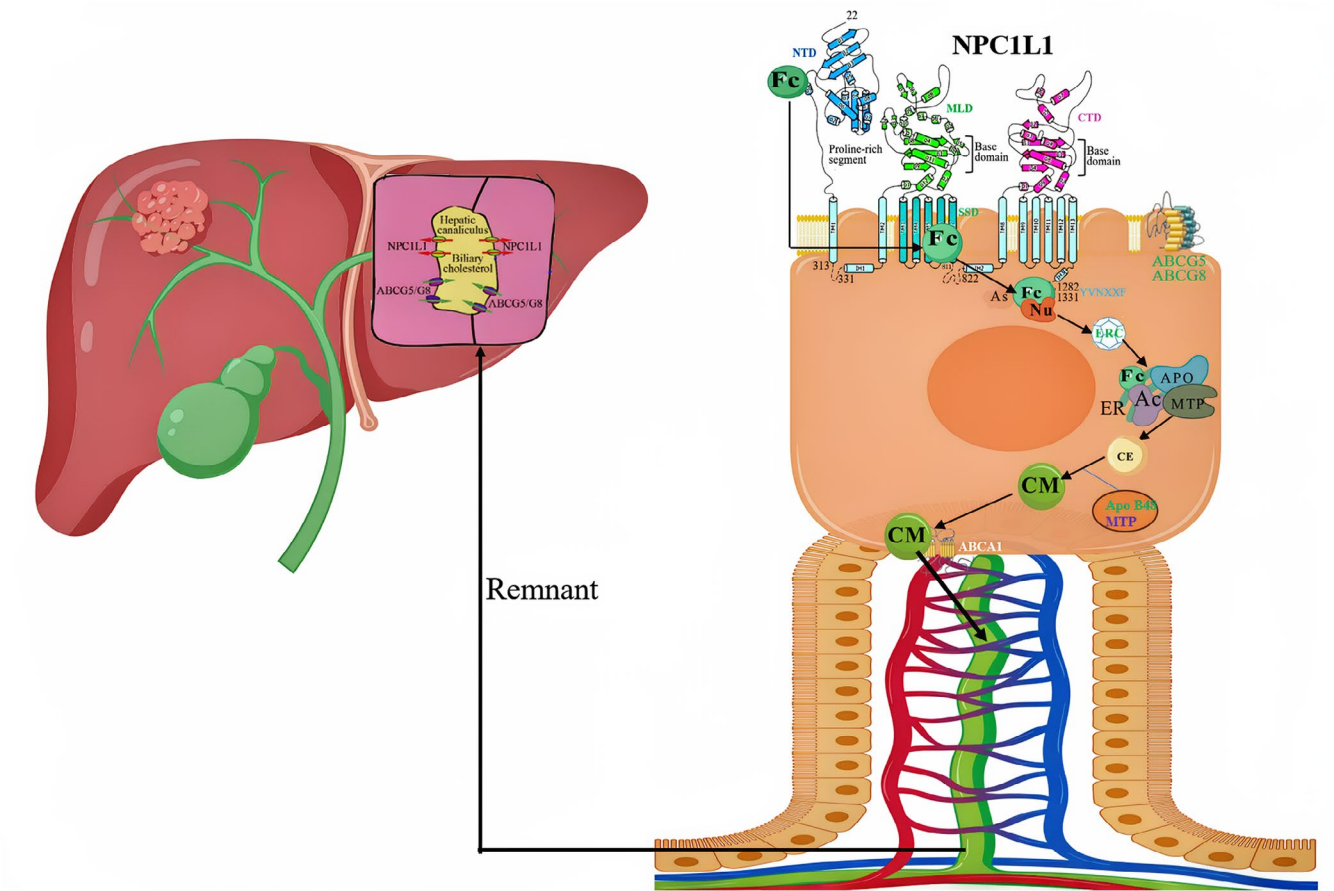
NPC1L1‐mediated exogenous uptake of cholesterol in the small intestine. Ac, ACAT2; As, aster; ce, cholesterol ester; CM, chylomicrons; ER, endoplasmic reticulum; ERC, endocytic recycling compartment; Fc, free cholesterol; MTP, microsomal triglyceride transfer protein; Nu, numb.

Thus, when dietary cholesterol is emulsified and digested in the small intestine, it is converted into free cholesterol, which directly binds to the NTD domain of NPC1L1 on the enterocyte plasma membrane (PM). The cholesterol is subsequently transferred to the SSD region of NPC1L1, inducing a conformational change in the SSD that exposes the C‐terminal endocytosis signal peptide YVNXXF. This peptide binds to the intracellular Numb and recruits clathrin to facilitate the endocytosis of free cholesterol [32]. This process triggers the recruitment of Aster protein to the PM, where Aster mediates the non‐vesicular transport of cholesterol to the endoplasmic reticulum (ER) [33]. Free cholesterol is then converted into cholesteryl esters by acyl‐CoA cholesterol acyltransferase 2 (ACAT2) in the ER. Along with triglycerides, some unesterified cholesterol, apolipoprotein B48, and microsomal triglyceride transfer protein (MTP), the cholesteryl esters are incorporated into nascent chylomicrons (CM) [34]. Cholesterol esters are secreted into the interstitial space as CM or HDL, migrate to the lamina propria, enter the central lacteals, and then converge into the thoracic duct (Figure 1). At the left venous angle of the neck, the cholesterol is returned to the liver for metabolism [20]. Unesterified cholesterol may be pumped back into the intestinal lumen by the apically located ATP‐binding cassette transporters G5/G8 (ABCG5/ABCG8) heterodimer or may be transported to the basolateral membrane of enterocytes [35, 36], where it is mediated by ABCA1 to facilitate the biogenesis of HDL [37, 38]. In addition to exogenous acquisition, cholesterol in the serum can also be synthesized de novo within cells.

### 2.2 Endogenous Synthesis

**FIGURE 2:**
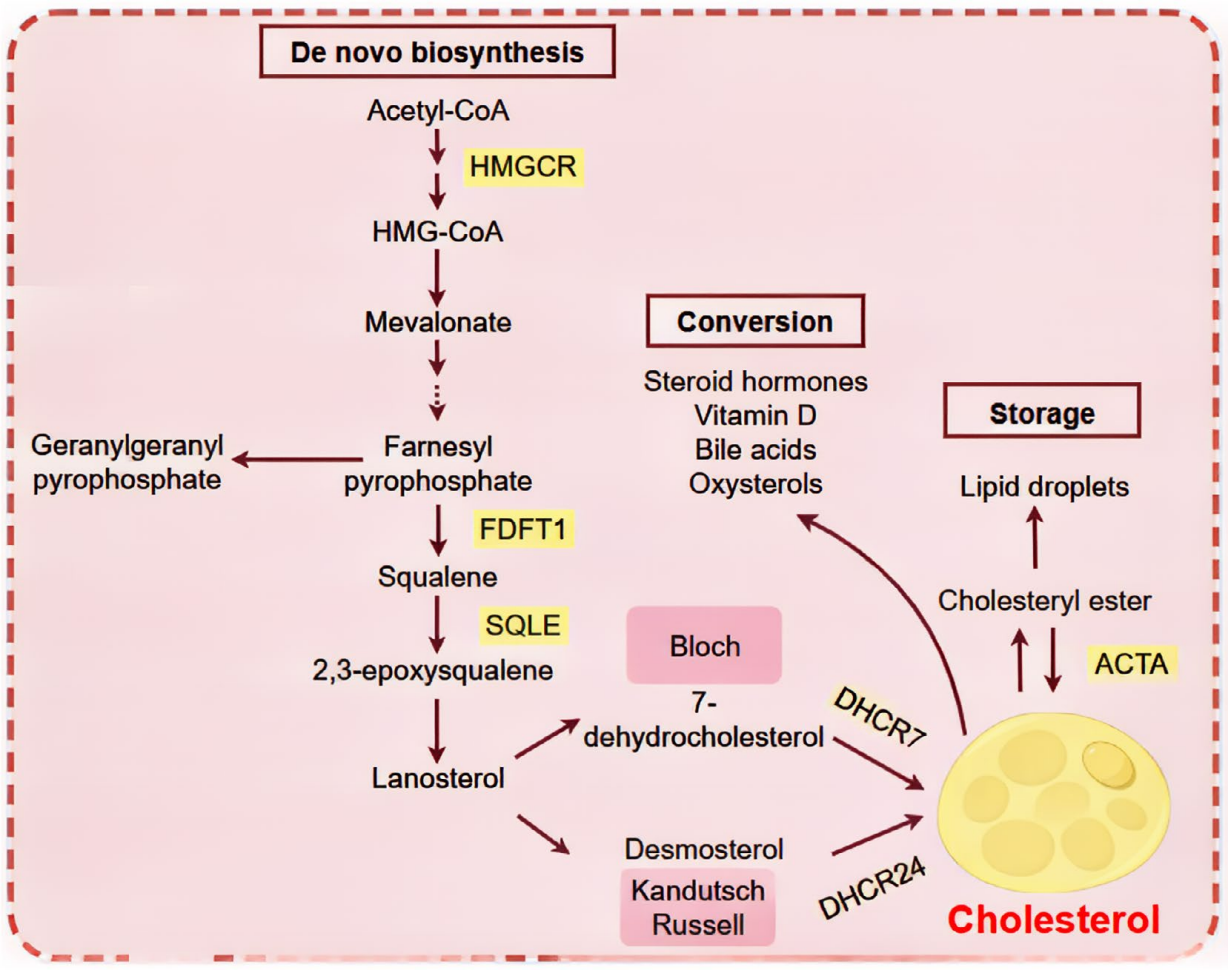
De novo biosynthesis, conversion, and storage of cholesterol in organisms.

The liver plays a central role in cholesterol synthesis. Cholesterol synthesis is a multi‐step biochemical process that requires substantial energy expenditure, involving 18 acetyl‐CoA molecules, 36 ATP molecules, 16 NADPH molecules, and 11 oxygen molecules to produce one molecule of cholesterol [3]. The conversion of acetyl‐CoA into cholesterol entails nearly 30 enzymatic steps, including the mevalonate pathway, squalene synthesis, and its subsequent transformations [39]. In the cytoplasm, two acetyl‐CoA molecules combine to form acetoacetyl‐CoA, which then reacts with another acetyl‐CoA molecule to generate 3‐hydroxy‐3‐methylglutaryl‐CoA (HMG‐CoA). HMG‐CoA is subsequently converted into the key intermediate mevalonate by the enzyme 3‐hydroxy‐3‐methylglutaryl‐coenzyme A reductase (HMGCR), which catalyzes the mevalonate pathway [1]. Following this, mevalonate is converted into farnesyl pyrophosphate (FPP) through a series of enzymatic reactions involving phosphomevalonate kinase (PMVK) and mevalonate diphosphate decarboxylase (MVD). FPP serves as a precursor for cholesterol sterols and various non‐steroidal isoprenoid compounds [40]. FPP then undergoes further transformation under the action of squalene epoxidase (SQLE, also known as squalene monooxygenase), which converts it into 2,3‐epoxysqualene, thereby initiating sterol synthesis [41]. Cholesterol is subsequently synthesized through the action of 24‐dehydrocholesterol reductase (DHCR24) and 7‐dehydrocholesterol reductase (DHCR7) [42, 43]. Cholesterol is esterified by acyl‐CoA cholesterol acyltransferase (ACAT) and is ultimately stored in lipid droplets within the cell (Figure 2).

The endogenous synthesis of cholesterol is influenced by various enzymes, which, in turn, impact multiple diseases [44]. Therefore, the expression and degradation of these enzymes are regulated by specific proteins. Understanding this process can provide deeper insights into the pathological mechanisms of cholesterol metabolism disorders.

### 2.3 Regulation of Cholesterol Synthesis and Intake

#### 2.3.1 HMGCR

**FIGURE 3:**
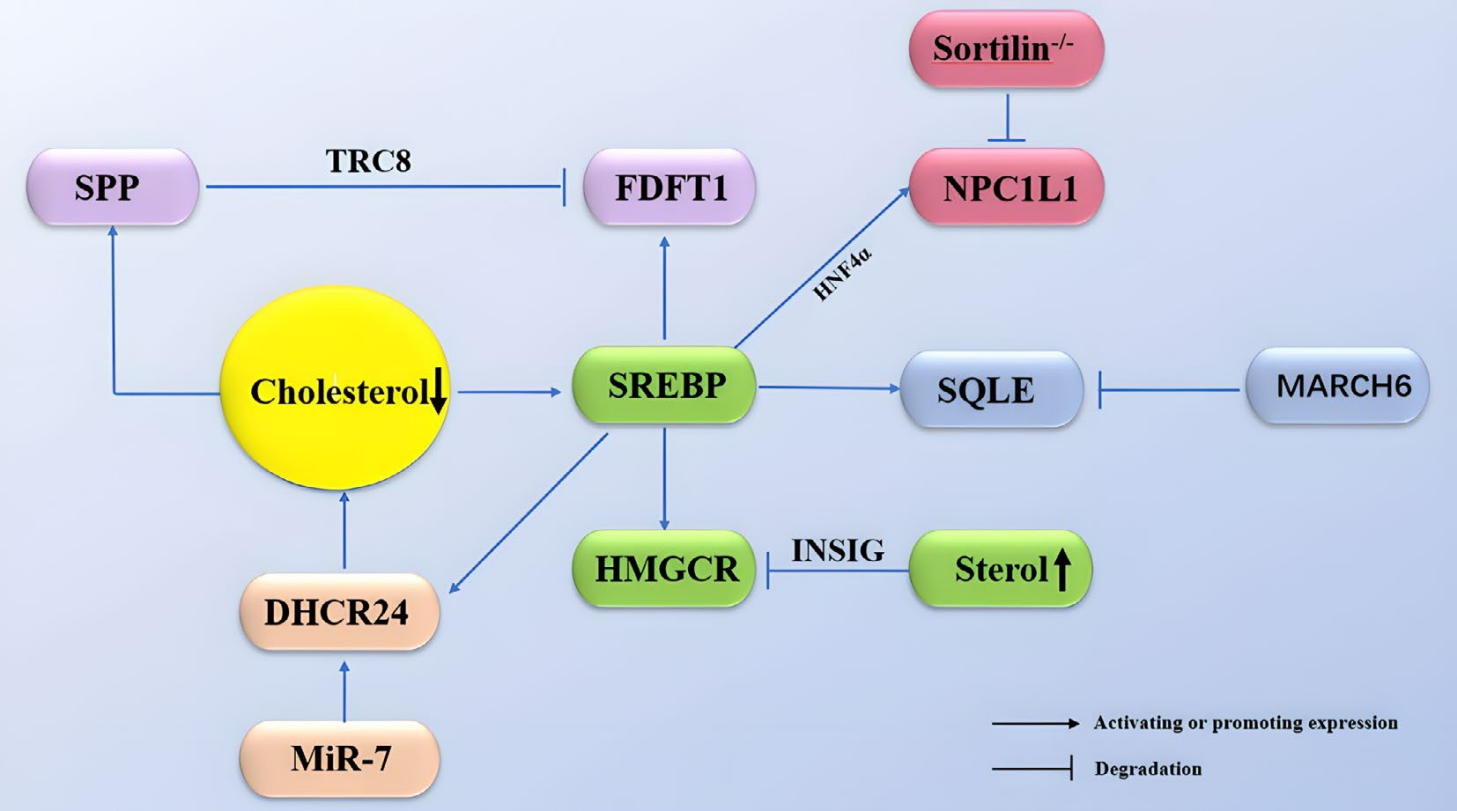
Regulatory mechanisms involved in cholesterol uptake and synthesis.

In the mevalonate pathway, HMGCR is responsible for reducing HMG‐CoA to mevalonate. Mammalian HMGCR is a glycoprotein localized in the ER, consisting of a hydrophobic NTD that spans the membrane eight times and a large, soluble C‐terminal domain that extends into the cytoplasm. The N‐terminal TMDs 2–6 form the SSD of HMGCR, making it sensitive to sterol levels in the ER membrane. The C‐terminal domain in the cytoplasm utilizes two molecules of NADPH as reducing agents to convert HMG‐CoA into mevalonate [45]. When intracellular sterol concentrations are low, the HMGCR gene is activated by nSREBP2, initiating the de novo synthesis of cholesterol [46] (Figure 3). The deubiquitinating enzyme ubiquitin‐specific peptidase 20 (USP20) stabilizes HMGCR in the fed state. Gene deletion or pharmacological inhibition of USP20 significantly reduces diet‐induced weight gain, lowers serum and hepatic lipid levels, improves insulin sensitivity, and increases energy expenditure [47]. The degradation of HMGCR is primarily induced by oxysterols and lanosterol, while cholesterol has a relatively weaker effect on promoting HMGCR degradation [48]. INSIG is essential for the degradation of HMGCR induced by sterols. When oxysterols and lanosterol accumulate inside the cell, INSIG1 is activated to bind to the membrane domain of HMGCR [48, 49], triggering HMGCR ubiquitination, followed by its degradation via the proteasome [50].

#### 2.3.2 FDFT1

Farnesyl‐diphosphate farnesyltransferase 1 (FDFT1, also known as squalene synthase, SQS) is a key enzyme in the endogenous cholesterol biosynthesis pathway, catalyzing the condensation of two FPP molecules to produce squalene [51]. This enzyme consists of 416 amino acids, has a molecular weight of 47 kDa, and is almost exclusively localized in the ER [52]. The expression of FDFT1 is transcriptionally regulated by SREBP (Figure 3). When intracellular cholesterol levels decrease, the SSD of SREBP detects this change and initiates the transcription of FDFT1, thereby increasing intracellular cholesterol levels [53]. The ER membrane protein complex (EMC) facilitates the insertion of the weakly hydrophobic tail‐anchor (TA) of FDFT1 into the ER membrane, ensuring adequate flux through the sterol biosynthesis pathway. Additionally, EMC promotes the biogenesis of sterol‐O‐acyltransferase 1 (SOAT1), which enables cells to store free cholesterol as inert cholesterol esters [54]. Under high cholesterol conditions, signal peptide peptidase (SPP) cleaves SQS. This intramembrane cleavage releases SQS from the ER membrane, marking it for degradation by the proteasome. This regulatory mechanism is controlled by the E3 ubiquitin ligase TRC8 [55].

#### 2.3.3 SQLE

SQLE regulates cholesterol biosynthesis by catalyzing the conversion of squalene into 2,3‐oxidosqualene [56]. In human cells, the gene encoding SQLE is located on chromosomal region 8q24.1 [57]. SQLE is a direct target of SREBP2, and its encoded protein contains a SSD that mediates its degradation via the proteasome. Similar to HMGCR, the activity of SQLE is finely regulated by intracellular cholesterol levels through a feedback mechanism, making it the second rate‐limiting step in the cholesterol biosynthesis pathway [58]. A study by J. M. Tan et al. [59] identified UBE2J2 as a critical cofactor of MARCH6, facilitating cholesterol‐induced ubiquitination and subsequent proteasomal degradation of SQLE (Figure 3). Recent research further suggests that SQLE degradation also depends on the formation of a transmembrane channel by Doa10/MARCH6, as well as the interaction between its RING domain and the lipid‐binding site [60].

#### 2.3.4 DHCR24

3β‐Hydroxysterol‐Δ24 reductase (DHCR24, also known as Seladin‐1) is the final enzyme in the cholesterol biosynthesis pathway, playing a critical role in lipid raft formation and catalyzing the reduction of the Δ24 double bond in sterol precursors to produce cholesterol [61, 62]. The DHCR24 gene is located on chromosome 1p32.3, spanning 46.4 kb and consisting of 8 introns and 9 exons. It encodes 1551 amino acids and is highly expressed in the adrenal gland, spinal cord, medulla oblongata, liver, lungs, spleen, and prostate [63, 64, 65]. In the liver of mice and cultured human hepatocytes, activation of the Constitutive Androstane Receptor (CAR) increases the mRNA levels of DHCR24 in mice and DHCR24 in humans, leading to elevated serum cholesterol and hepatic cholesterol biosynthesis [66]. Oxysterols derived from the mevalonate pathway, including 25‐hydroxycholesterol (25HC) and 24,25‐epoxycholesterol (24,25EC), reduce DHCR24 expression. The expression of DHCR24 is primarily regulated by SREBP‐2, which binds to two sterol regulatory elements (SREs) in the DHCR24 gene promoter to initiate transcription [67]. Additionally, MiR‐7, a member of the microRNA (miRNA) family, regulates cholesterol biosynthesis by post‐transcriptionally controlling DHCR24 expression [68] (Figure 3).

#### 2.3.5 SREBP

Throughout the preceding discussion, the regulatory role of SREBPs in cholesterol biosynthesis has been repeatedly emphasized. SREBPs are members of the membrane‐bound transcription factor family and are classified as basic helix–loop–helix leucine zipper (bHLH‐LZ) transcription factors. The two major family members, SREBP1 and SREBP2, play essential roles in regulating the expression of lipogenic genes and cholesterol biosynthesis genes, respectively [69]. The SREBF1 gene is located on chromosome 17, with a coding region of 3534 bp, comprising 20 exons and encoding 1177 amino acids. In contrast, the SREBF2 gene is located on chromosome 22, with a coding region of 3426 bp, consisting of 19 exons and encoding 1141 amino acids [70]. Sterol regulatory element‐binding protein 1 (SREBP1) has two splicing isoforms: SREBP1a and SREBP1c. Together with SREBP2, these isoforms exert distinct transcriptional effects, depending on whether their target genes contain SREs, SRE‐like sequences, or E‐box motifs. Notably, SREBP1a strongly activates genes containing SREs or SRE‐like sequences but is less effective in activating genes with E‐box motifs. In contrast, SREBP1c can activate both E‐box sequences and SRE‐like sequences, while SREBP2 strongly activates SREs, weakly activates SRE‐like sequences, and does not activate E‐box sequences [71]. The regulation of SREBP1 is primarily controlled by caloric restriction [72], whereas SREBP2 is activated by thyroid hormones and through autoregulation [73]. SREBP2 must be translocated from the ER to the Golgi apparatus to become active. Sequential cleavage by site‐1 protease (S1P) and site‐2 protease (S2P) releases the N‐terminal fragment of SREBP2 from the ER membrane. After processing in the Golgi, the resulting nuclear SREBP2 (nSREBP2) enters the nucleus as a homodimer, where it binds to SRE sequences within the promoters of target genes, including HMGCR and SQLE, thereby upregulating their transcription [74] (Figure 3). SCAP is a polytopic escort protein localized in the ER, which facilitates the transport of inactive SREBP precursors from the ER to the Golgi [75]. Specifically, under conditions of sufficient cholesterol and oxysterol, SREBP2 is retained in the ER by directly binding to SCAP, which, in turn, is anchored by insulin‐induced gene proteins (INSIGs). When intracellular sterol levels decline, SCAP undergoes a conformational change, dissociating from INSIG and allowing the SCAP/SREBP2 complex to translocate to the Golgi, where SREBP2 undergoes cleavage (Figure 3). Once activated, SREBP2 moves to the nucleus, where it acts as a transcription factor [76]. Intracellular cholesterol can be associated with transporters, particularly ATP‐binding cassette transporters ABCA1 and ABCG1, to facilitate cholesterol efflux, a key step in the maintenance of cholesterol homeostasis. However, miR‐33, which is co‐expressed with SREBPs, inhibits the activity of these transporters, thereby preventing cholesterol efflux [77, 78]. Acetyl‐CoA synthetase, a downstream effector of SREBPs, is critical for lipid biosynthesis and promotes the release of reactive oxygen species (ROS), nitric oxide (NO), and prostaglandin E2 (PGE2) [79].

The regulation of serum cholesterol levels is influenced not only by endogenous cholesterol synthesis but also by exogenous cholesterol uptake. The latter is primarily controlled through the regulation of NPC1L1.

#### 2.3.6 NPC1L1

The protein structure of NPC1L1 has been elucidated in the Exogenous Acquisition section and is therefore not repeated here. NPC1L1 can bind varying numbers of cholesterol molecules through its SSD to respond to fluctuations in cholesterol levels [30]. NPC1L1 functions in intestinal cholesterol uptake only when it is translocated from the endocytic recycling compartment (ERC) to the PM. When cholesterol accumulates within the cell, NPC1L1 remains in the ERC, preventing the absorption of intestinal cholesterol [80]. In contrast, when bile acid (BA) synthesis increases, leading to reduced cholesterol levels in the ERC, the A1272LAL motif of NPC1L1 interacts with cholesterol‐enriched membranes, exposing its Q1277KR motif. This interaction recruits LIMA1 and induces NPC1L1 translocation to the PM. Thus, BAs facilitate NPC1L1 recycling to the cell surface, enhancing cholesterol absorption [81]. In mice fed a high‐cholesterol diet, intestinal NPC1l1 expression is significantly reduced [25, 82]. Similar to HMGCR, the human NPC1L1 gene contains two SREs and can be activated by sterol regulatory element‐binding protein 2 (SREBP2) [83, 84]. Additionally, hepatocyte nuclear factor 4α (HNF4α) enhances SREBP2‐mediated NPC1L1 activation [85] (Figure 3). In HepG2 cells, the peroxisome proliferator‐activated receptor α (PPARα)‐retinoid X receptor α (RXRα) nuclear receptor complex upregulates NPC1L1 transcription [86]. Conversely, the activation of liver X receptors (LXRs) and the absence of sortilin downregulate NPC1L1 expression [87, 88]. Hepatic NPC1L1 prevents excessive cholesterol loss by reabsorbing cholesterol from canalicular bile [80], whereas ABCG5/ABCG8 mediates cholesterol efflux from hepatocytes into bile [89]. Mice engineered to overexpress hepatic NPC1L1 (NPC1L1^hepatic‐OE^ mice) using adeno‐associated virus (AAV) gene delivery demonstrated that a lithogenic diet induces hepatic NPC1L1 ubiquitination and degradation through the FGF15‐FGFR4 pathway [90]. The specific inhibitor of NPC1L1, ezetimibe, inhibits NPC1L1 activity in both intestinal epithelial cells and hepatocytes, thereby reducing cholesterol absorption [91]. Recent studies have shown that in mice, diosgenin (DG) downregulates Npc1l1 expression by inhibiting STAT3 phosphorylation, thereby reducing cholesterol absorption in the intestine and Caco‐2 cells [92].

Imbalances in cholesterol regulation significantly contribute to the onset of various diseases, including heart disease, neurological disorders, and cancer [44]. In particular, the therapeutic strategy of targeting cholesterol metabolism for cancer treatment has been extensively tested in clinical settings in recent years [93]. The following section will focus on the relationship between dysregulated cholesterol metabolism and cancer progression, as well as its role in tumor immunity.

## 3 Regulatory Components in Cholesterol Metabolism and the Role in Cancer

### 3.1 SREBPs

SREBPs are key regulators of cholesterol metabolism, playing a critical role not only in metabolic diseases but also as crucial factors in tumor development [73]. SREBPs contribute to the establishment of energy and lipid reserves, immune status, and the inflammatory environment in tumor cells, acting as a defense barrier that facilitates the malignant growth of tumor cells [94]. The liver is the central organ for cholesterol metabolism, and as such, dysregulation of cholesterol metabolism is closely associated with the development and progression of HCC. Andreas Koeberle and colleagues have provided an excellent review on the role of SREBP1 in the pathogenesis of HCC [95], focusing on the following aspects: First, high endogenous expression of SREBP1 is linked to poor prognosis and low survival rates in HCC. The use of SREBP1 inhibitors, such as Betulin and SI‐1, has shown therapeutic effects on xenografted HCC [96, 97]. In mouse models of liver cancer, p53 induces the expression of the ABCA1 cholesterol transporter gene via transcription, blocking the activation of SREBP‐2, the main transcriptional regulator of this pathway, thus inhibiting HCC development [98]. Jianjun Liu and colleagues found that CBF (Cinobufotalin) selectively inhibits SREBP1 expression and interacts with SREBP1 to prevent its binding to SREs, thereby inhibiting the expression of lipogenic enzymes and reducing tumor volume and weight in tumor‐bearing mice [99]. Through bioinformatics analysis and immunohistochemistry, it was found that NAD(P)H quinone oxidoreductase‐1 (NQO1), through the NQO1/p53/SREBP1 axis, induces the transcriptional activity of SREBP1, promoting HCC progression and metastasis [100]. Zhi‐Qiang Meng and colleagues performed transcriptomic and metabolomic analyses and found that Bufalin, a classic Na‐K‐ATPase (NKA) inhibitor, inhibits HCC lipogenesis and tumorigenesis by regulating the ATP1A1/CA2 axis to downregulate SREBP1/FASN/ACLY [101]. Moreover, the lack of Slc25a47 (a mitochondrial carrier protein) inhibits AMPKα phosphorylation, leading to the accumulation of nuclear SREBPs, enhanced fatty acid and cholesterol biosynthesis, and promoting HCC tumorigenesis and development [102]. Recent studies have shown that Ficolin 3 (FCN3), a component of the complement system, directly binds to insulin receptor β (IR‐β) and its precursor pro‐IR, inhibiting the cleavage of pro‐IR and the phosphorylation of IR‐β, ultimately leading to the inactivation of IR‐β. Inactivation of IR‐β suppresses SREBP1c expression, downregulating monounsaturated fatty acid levels within HCC cells, making them sensitive to ferritin deposition, thereby inhibiting the tumorigenesis and progression of primary HCC and subcutaneous HCC xenografts [103]. These studies demonstrate that inhibiting SREBP can suppress the occurrence and progression of HCC through multiple pathways.

Another tumor closely related to SREBP is glioblastoma (GBM). EGFR mediates SREBP1 cleavage and nuclear translocation via Akt, and the constitutively active mutant EGFRvIII sensitizes glioblastoma xenografts in mice to cell death [104]. The authors used avasimibe to inhibit sterol O‐acyltransferase (SOAT) and shRNA lentivirus to knock down SOAT1 expression, inhibiting SREBP1 activity, significantly reducing cholesteryl ester (ce) levels in GBM cells and decreasing lipid droplet (LD) formation [105]. Qianxue Chen and colleagues, through transcriptomic sequencing, in vivo and in vitro experiments, found that the SREBP‐specific inhibitor fatostatin induces ferroptosis in glioblastoma by inhibiting the AKT/mTORC1/GPX4 signaling pathway [106]. In the same year, Xiuxing Wang and colleagues performed in silico analysis of glioblastoma patients and found that SREBP2 promotes cholesterol biosynthesis in glioblastoma stem cells (GSCs), inducing cholesterol uptake at the tumor edge [107]. Recent studies show that glioblastoma develops resistance to pimozide by increasing glutamine consumption and lipogenesis. SREBP1 upregulates the expression of the glutamine transporter ASCT2, and pharmacological targeting of ASCT2 or glutaminase, in combination with pimozide, disrupts this cycle, inducing significant mitochondrial damage and oxidative stress, leading to GBM cell death both in vitro and in vivo [108]. In addition to HCC and GBM, the inhibition of SREBP has therapeutic effects in esophageal squamous cell carcinoma [109], gastric cancer [110], colon cancer [111, 112], pancreatic cancer [113, 114], clear cell renal cell carcinoma [115], breast cancer (BC) [116, 117, 118], and B‐cell lymphoma [119] (for detailed information, refer to Table 1).

**TABLE 1:** The role of enzymes involved in cholesterol metabolism in cancer development.

Target	Inhibitor	Tumor type	Functional mechanism	Conclusion	References
SREBP2	Data not available‌‌	HCC	p53 induces the expression of the AbcA1 through transcriptional activation, thereby blocking the activation of SREBP2	Inhibited the development of HCC	[98]
SREBP1	Cinobufotalin	HCC	Cinobufotalin inhibits the expression of SREBP1, preventing its binding to sterol SREs	Reduced the tumor volume and weight	[99]
SREBP1	Data not available‌‌	HCC	NQO1 can induce the transcriptional activity of SREBP1 through the NQO1/p53/SREBP1 axis	Facilitated the progression and metastasis of HCC	[100]
SREBP1	Bufalin	HCC	Bufalin regulates the ATP1A1/CA2 axis to downregulate SREBP‐1/FASN/ACLY	Inhibited lipogenesis and tumorigenesis in HCC	[101]
SREBPs	Data not available‌‌	HCC	The deficiency of Slc25a47 inhibits AMPKα phosphorylation, leading to an increased accumulation of nuclear SREBPs	Promoted the tumorigenesis and progression of HCC	[102]
SREBP1c	Data not available‌‌	HCC	Ficolin 3 binds to the IR‐β and its precursor, inhibiting the cleavage of pro‐IR and the phosphorylation of IR‐β, leading to the inactivation of IR‐β and suppression of SREBP1c expression	Inhibited tumorigenesis and progression in primary HCC and subcutaneous HCC xenografts	[103]
SREBP1	Data not available‌‌	GBM	EGFR mediates the cleavage and nuclear translocation of SREBP‐1 through Akt	Made GBM xenografts sensitive to cell death	[104]
SOAT	Avasimibe	GBM	Inhibition or knockdown of SOAT1 suppressed the activity of SREBP‐1, significantly reducing‌ the levels of CE	Inhibited GBM growth and prolonged survival	[105]
SREBPs	Fatostatin	GBM	Inhibition of the AKT/mTORC1/GPX4 signaling pathway in GBM induced ferroptosis	Ferroptosis occurs in GBM	[106]
SREBP2	Data not available‌‌	GBM	SREBP2 promotes cholesterol biosynthesis in GBM stem cells	Promoted tumor cell proliferation	[107]
SREBP1	Data not available‌‌	GBM	SREBP‐1 upregulates the expression of ASCT2	GBM cell death	[108]
LPCAT1	Data not available‌‌	ESCC	LPCAT1 promotes the activation of HER and PI3K, facilitating the nuclear translocation of SREBP‐1 with SP1/SREBF2	Knockdown of LPCAT1 inhibited tumor cell proliferation, invasion, and migration	[109]
SREBP1c	HCPA	GC	Activation of SREBP‐1c expression led to the upregulation of SCD1 and FASN expression	HCPA specifically inhibits cell proliferation and invasiveness in GC	[110]
SREBPs	Data not available‌‌	CC	Knockout of SREBP1 or SREBP2 reduces mitochondrial respiration, glycolysis, and fatty acid oxidation	Inhibited the growth of xenograft tumors	[111]
FUT2	Data not available‌‌	CRC	FUT2 promotes the nuclear translocation of YAP/TAZ and reprograms fatty acids via SREBP‐1	Promoted the proliferation and metastasis of CRC cells	[112]
SREBP1	Timosaponin A3	PC	TA3 inhibits SREBP‐1, leading to a decrease in the expression of FASN and ACC	Reduced lipogenesis and growth in BxPC‐3 cells	[113]
MED15	Data not available‌‌	ccRCC	MED15 promotes the activation of SREBP1 and SREBP2 through the LK1/AKT axis	Promoted the development of ccRCC	[115]
SREBP1a	Data not available‌‌	BC	The residue Y267 of p54(nrb) enhances the stability of nuclear SREBP‐1a protein	Promoted the progression of breast cancer	[116]
SREBP1	Data not available‌‌	BC	SREBP1/SCD1‐mediated PI3K‐AKT–mTOR signaling hyperactivation	Protects cancer cells from ROS and ferroptotic cell death	[117]
SREBP2	Pitavastatin	TNBC	The combination of pitavastatin and the AKT inhibitor GDC‐0068 leads to dysregulated cholesterol transport	TNBC exhibits susceptibility to the combination therapy of GDC‐0068 and pitavastatin	[118]
SREBP	Fatostatin	BCL	Activates the mTORC1‐pS6 pathway	Inhibited the proliferation, and lipid raft formation of B‐cell lymphoma	[119]
HMGCR	Simvastatin	HCC	The cholesterol‐TAZ‐TEAD2‐ANLN/KIF23 pathway	Reduced proliferation of HCC cells	[120]
TEAD	VT104/VT107				
PCSK9	R‐IMPP	HCC	Data not available‌‌	Decreased proliferation of HCC cells	[121]
HMGCR	Simvastatin				
HMGCR	Simvastatin	HCC	Activation of the Hedgehog signaling pathway	Promoted the progression of HCC	[122]
HMGCR	Simvastatin	TNBC	Fe3O4@PCBMA‐SIM nanoparticles inhibit the expression of HMGCR	Induced ferroptosis in TNBC cells	[123]
HMGCR	Lovastatin	BC	The combination therapy of lovastatin and paclitaxel enhances the activity of CD8^+^ T cells	Enhanced the ability of CD8^+^ T cells, improving the prognosis of BC	[124]
HMGCR	Atorvastatin/simvastatin	BC	p140Cap positively regulates HMGCR through transcriptional and post‐translational mechanisms	Reduction‌ the viability of BC cells	[125]
HMGCR	Data not available‌‌	EOC	Data not available‌‌	HMGCR inhibition is associated with a lower risk of EOC	[126]
HMGCR	Data not available‌‌	LC	SIAH1 can ubiquitinate and modify HMGCR, inhibiting the activity of efflux proteins by regulating cholesterol synthesis	It can delay the growth of LC cells and enhance the efficacy of cisplatin	[127]
HMGCR	Data not available‌‌	GLI	hnRNPA2B1 induces HMGCR by stabilizing SREBP2 mRNA	Targeting hnRNPA2B1 and HMGCR demonstrates significant anti‐tumor effects	[128]
ATG7	ATG7‐IN‐1	CRC	Inhibition of ATG7 can restore the levels of MHC‐I, improving antigen presentation and anti‐tumor T cell responses by activating the ROS/NF‐κB pathway	The combination of ATG7‐IN‐1 and statin use can enhance the therapeutic efficacy of anti‐PD‐1 in CRC	[129]
HMGCR	Atorvastatin				
HMGCR	Data not available‌‌	ETP‐ALL	Inhibition of AKT1 signaling and reduction‌ of MYC expression	Inhibition of the growth of leukemia cells	[130]
FDFT1	Data not available‌‌	CRC	FDFT1 negatively regulates the AKT/mTOR/HIF1α signaling pathway	Inhibit the proliferation of tumor cells	[131]
FDFT1	Data not available‌‌	Lymphomas	Squalene elevation protects the cell membrane from oxidative stress by preventing lipid peroxidation, thus inhibiting ferroptosis	Promoted the development of the tumor	[132]
EBP	TASINs	CRC	TASINs induce cancer cell death by inhibiting EBP and depleting downstream sterols	EBP is a target in APC mutant colorectal cancer	[133]
EBP	cis‐33	PCa	Competitively binding to EBP‐related sigma receptors inhibits the proliferation of r PC‐3 cell line	This class of novel EBP inhibitors	[134]
EBP	Data not available‌‌	ALCL	NPM‐ALK activates EBP	The RNA and protein levels of EBP are overexpressed	[135]
SQLE	NB‐598	CRC	SQLE inhibition lowers calcitriol and CYP24A1, raises Ca2+ levels, and suppresses MAPK signaling	SQLE is highly upregulated in CRC and is associated with poor prognosis	[136]
SQLE	Data not available‌‌	CRC	SQLE deletion causes cholesterol buildup, activating the β‐catenin pathway and inactivating p53	SQLE is a therapeutic target for CRC	[137]
SQLE	Data not available‌‌	PCa	PTEN/p53 deficiency upregulates SQLE transcription by activating SREBP2 and inhibits the PI3K/Akt/GSK3β pathway	Promote tumor cell growth and survival	[138]
SQLE	Data not available‌‌	HCC	Activation of the TGF‐β/SMAD signaling pathway	Enhance HCC growth	[139]
SQLE	Terbinafine	HNSCC	SQLE inactivates raft‐localized Akt, decreases GSK‐3β phosphorylation, and increases c‐Myc phosphorylation, leading to c‐Myc destabilization	Promote the occurrence and progression of HNSCC	[140]
LPCAT1	Data not available‌‌	ESCC	LPCAT1 enhances SQLE expression by facilitating the activation of PI3K	LPCAT1 alters cholesterol metabolism in ESCC and is a potential therapeutic target	[109]
SOX9	Data not available‌‌	DLBCL	Knockdown of SOX9 leads to a reduction DHCR24 levels	The SOX9‐DHCR24‐cholesterol biosynthesis axis is a novel target for DLbcL	[141]
SRSF3	SFI003	CRC	The SRSF3/DHCR24/ROS pathway induces apoptosis in CRC cells	SRSF3 is a novel therapeutic target for CRC	[142]
DHCR24	SH42	Atherosclerosis	SH42 is LXRα‐dependent	Prevent diet‐induced hepatic steatosis and inflammation	[143]
DHCR24	Data not available‌‌	HCC	DHCR24 inhibits p53 activation by enhancing its binding to MDM2, blocking its protective function	DHCR24 is an important therapeutic target for HCV‐associated HCC	[144]
NPC1L1	Ezetimibe	HCC	NPC1L1 inhibits the PI3K/AKT/mTOR signaling pathway	Ezetimibe effectively inhibits the proliferation of HCC cells	[145]
PCSK9	Data not available‌‌	CRC	PCSK9 promotes CRC cell proliferation, migration, and invasion via EMT and the PI3K/AKT pathway	Inhibition of PCSK9 effectively suppresses the progression of CRC	[146]
PCSK9	PF‐0644846 /R‐IMPP	CRC	GGPP promotes the development of APC/KRAS‐mutant CRC by activating the KRAS/MEK/ERK signaling pathway	Inhibition of PCSK9 suppresses the growth of APC/KRAS‐mutant CRC cells both in vitro and in vivo	[147]
PCSK9	Evolocumab	CRLC	Inhibition of PCSK9 in vivo downregulates the expression levels of RUNX1 in CRLC cancer cells	Inhibition of PCSK9 is an effective therapeutic approach to CRLC	[148]
PCSK9	Data not available‌‌	HCC	Through the FASN/Bax/Bcl‐2/Caspase9/Caspase3	PCSK9 inhibited apoptosis of HCC	[149]
PCSK9	Data not available‌‌	HCC	PCSK9 interacts with GSTP1, disrupting GSTP1 dimers and inactivating the JNK pathway	PCSK9 promotes the growth of HCC	[150]
PCSK9	CPPtat‐1	HCC	ZDHHC16 S‐palmitoylates PCSK9 at C600, enhancing its affinity for PTEN, leading to PTEN degradation and AKT activation	PCSK9 upregulation induces AKT‐S473 phosphorylation, promoting sorafenib resistance in HCC.	[151]
PCSK9	Data not available‌‌	HCC	PCSK9 inhibition disrupts the p62/Keap1/Nrf2 axis, impairing tumor metabolism and increasing cancer cell vulnerability to iron‐induced lipid peroxidation	Anti‐PCSK9 strategies are effective therapies for the treatment of HCC	[152]
PCSK9	Flubendazole	HCC	Flubendazole inhibits PCSK9‐induced Hedgehog activation by reducing PCSK9, downregulating SMO and Gli1	The combination of flubendazole and lenvatinib enhances the anticancer effects of lenvatinib	[153]
PCSK9	PF‐06446846	HCC	PCSK9 promotes LDL receptor (LDLR)‐mediated mTORC1 signaling activation in CD8^+^ T cells	LDLR absence impairs mTORC1 signaling and CD8^+^ T cell antitumor function in HCC	[154]
PCSK9	Data not available‌‌	GC	PCSK9 upregulates HSP70 and promotes the MAPK pathway	High PCSK9 expression in GC correlates with progression and poor prognosis	[155]
PCSK9	Data not available‌‌	BC	PCSK9 targets the tumor‐associated LRP1 receptor to inhibit the metastasis‐promoting genes XAF1 and USP18	Promote pulmonary metastasis and increase metastatic potential	[156]
PCSK9	Data not available‌‌	PCa	PCSK9 affects radiation sensitivity via mitochondrial pathways	PCSK9 is a novel target for PCa therapy	[157]
PCSK9	pseurotin A(PS)	PCa	Regulation of the PCSK9‐LDLR axis	PS can inhibit PCa recurrence	[158]
PCSK9	Evolocumab/alirocumab	NMSC	Data not available‌‌	ASCVD patients taking PCSK9 inhibitors have a lower risk of developing NMSC	[159]

Abbreviations: ALCL, anaplastic large cell lymphoma; BC, breast cancer; BCL, B‐cell lymphoma; CC, colon cancer; ccRCC, clear cell renal cell carcinoma; CE, cholesterol esters; CPPtat‐1, PCSK9‐derived peptide; CRC, colorectal cancer; EOC, epithelial ovarian cancer; ESCC, esophageal squamous cell carcinoma; ETP‐ALL, early T‐cell precursor acute lymphoblastic leukemia; GBM, glioblastoma; GC, gastric cancer; GLI, glioma; Gli1, GLI family zinc finger 1; HCC, hepatocellular carcinoma; HCPA, high concentration palmitic acid; HER, epidermal growth factor receptor; HNSCC, head and neck squamous cell carcinoma; DLBCL, diffuse large B‐cell lymphoma; CRLC, colorectal cancer liver metastasis; NMSC, non‐melanoma skin cancer; HSP70, heat shock protein 70; IR‐β, insulin receptor β; LC, lung cancer; LD, lipid droplets; LRP1, low‐density lipoprotein receptor‐related protein 1; NQO1, NAD(P)H quinone oxidoreductase 1; PC, pancreatic cancer; PCa, prostate cancer; RUNX1, runt‐related transcription factor‐1; SIM, simvastatin; SREs, sterol regulatory elements; TNBC, triple‐negative breast cancer.

### 3.2 HMGCR

HMGCR is the first rate‐limiting enzyme in the cholesterol biosynthesis pathway via the mevalonate pathway. Overexpression of HMGCR promotes cancer cell growth and migration, while HMGCR knockdown inhibits tumorigenesis [160]. The liver is the central organ for cholesterol metabolism, and numerous studies have shown that HMGCR promotes the stemness and metastasis of HCC both in vitro and in vivo, and is associated with poor prognosis in HCC [122]. Therefore, targeting HMGCR is an effective strategy for HCC treatment. Statins, which are inhibitors of HMGCR, have been used in the treatment of various cancers, including HCC [120, 121, 122], BC [123, 124, 125], epithelial ovarian cancer [126], lung cancer [127], glioma [128], and colorectal cancer(CRC) [129], where the combination of statins and chemotherapy has been shown to enhance anti‐tumor effects [161] (Table 1). Early T‐cell precursor acute lymphoblastic leukemia (ETP‐ALL) shows an increase in the biosynthesis of phospholipids and sphingolipids, making it particularly sensitive to inhibition of the rate‐limiting enzyme HMGCR in the mevalonate pathway. This inhibition works by limiting cholesterol synthesis and disrupting the distal regulation of leukemia stem cell‐specific MYC enhancer chromatin, thereby suppressing oncogenic AKT1 signaling and reducing MYC expression [130].

### 3.3 FDFT1

Abnormal expression of FDFT1 occurs in a wide variety of cancers, making it a potential candidate biomarker and a novel target for cancer therapy [162]. In addition to its structural role in cholesterol formation, FDFT1's reactant, PSDP, also acts as a bioactive lipid. It inhibits phospholipase D and leukocyte functions, which in turn modulate cellular signaling and mitigate acute inflammation. By doing so, it reduces potential damage to the body's tissues. This action highlights PSDP's pivotal role in regulating inflammation, particularly within neutrophils [163]. FDFT1 is highly expressed in sphere‐forming stem cells of BC and neuroblastoma, which demonstrate a substantial capacity for both self‐renewal and differentiation, as well as resistance to cancer therapies [164, 165]. In CRC, FDFT1 functions as a tumor suppressor by negatively regulating the AKT/mTOR/HIF1α signaling pathway [131]. FDFT1 is not only an important gene for predicting the prognosis of CRC patients but has also been identified as a gene associated with ferroptosis [166, 167]. The expression of FDFT1 is significantly elevated during cell proliferation, indicating its involvement in proliferative signaling in cancer cells. Specifically, FDFT1 regulates the cell cycle, with its inhibition significantly hindering the synthesis phase. Additionally, FDFT1 activates the NF‐κB pathway, resulting in increased levels of anti‐apoptotic proteins such as Bcl‐xL, Bcl‐2, and Bax, while reducing levels of pro‐apoptotic proteins like caspase‐3, thus obstructing the apoptotic signaling pathway [168]. ML162 (a GPX4 inhibitor) treatment in FDFT1‐deficient ALK^+^ ALCL (ALK^+^ anaplastic large cell lymphoma) cells depleted squalene, damaging membrane PUFAs, key ferroptosis drivers, and generating lipid peroxides that induced ferroptosis [132]. Recent studies have also identified FDFT1 as a susceptibility gene for polycystic ovary syndrome [169].

### 3.4 EBP

EBP (Emopamil‐binding protein) is an ER membrane protein responsible for converting yeast sterols into dehydrosterols or platform sterols. These reaction products play essential roles in cholesterol biosynthesis, promoting cellular autophagy, and stimulating oligodendrocyte development. They proceed through two parallel but distinct pathways: the Bloch pathway and the Kandutsch/Russell pathway [170]. Inhibition of EBP leads to the accumulation of its substrate enzymes, sterols, and enzymatic enols, thereby promoting autophagy in tumor cells [171]. Notably, EBP exhibits an affinity for a variety of pharmacologically active substances, including mood enhancers, antipsychotic drugs, analgesics, cholesterol synthesis inhibitors, and anticancer agents [172]. The Hedgehog signaling pathway is crucial for vertebrate development and cancer formation, with its core involving Smoothened (SMO), a cell signal transducing protein. The attachment of cholesterol to a specific site on the human SMO protein (asparagine 95) is a key step, influenced by Hedgehog and another protein, PTCH1, activating SMO. Moreover, EBP, as an SMO‐interacting protein, inhibits the cholesteroylation of SMO and the activity of the Hh pathway when overexpressed. Conversely, EBP gene disruption enhances SMO cholesteroylation and its downstream signaling. Notably, EBP‐mediated inhibition of SMO cholesteroylation is independent of its isomerase activity and depends on the C‐terminal of EBP, which is essential for its binding to SMO [173]. In contrast, in CRC, TASIN (Truncated APC‐Selective Inhibitors) selectively targets APC gene‐mutant CRC cells. TASINs induce cell death by inhibiting EBP and depleting downstream sterols. Therefore, EBP serves as a target for APC‐mutant CRC [133]. EBP inhibitors have demonstrated beneficial inhibitory effects on the proliferation of the human prostate cancer PC‐3 cell line [134]. Additionally, in ALK‐positive anaplastic large‐cell lymphoma, EBP accumulation has been observed at both the mRNA and protein levels [135].

### 3.5 SQLE

SQLE overexpression reduced squalene, increased lipid ROS, and enhanced ALK^+^ ALCL cell sensitivity to GPX4 inhibitors (ML162, RSL3), inhibiting tumor growth [132]. SQLE catalyzes the conversion of squalene to 2,3‐oxidosqualene and has been identified as a critical driver of chemoresistance and tumorigenesis through cholesterol‐dependent pathways. SQLE is considered an oncogene that promotes carcinogenic signaling, with frequent reports of SQLE amplification and differential expression in cancer [174]. SQLE promotes CRC cell proliferation by inducing cell cycle progression and inhibiting apoptosis. In contrast, inhibition of SQLE reduces the levels of calcitriol (the active form of vitamin D3) and CYP24A1, which subsequently increases intracellular Ca2 +^+^ concentrations, suppresses MAPK signaling and inhibits CRC cell growth [136]. Cholesterol accumulation resulting from SQLE deletion activates the β‐catenin oncogenic pathway and inactivates the p53 tumor suppressor pathway, thus exacerbating CRC progression [137]. Moreover, the combination of the SQLE inhibitor terbinafine with oxaliplatin and 5‐fluorouracil synergistically inhibits CRC growth [175]. In prostate cancer, PTEN/p53 deficiencies activate SREBP2 transcription, upregulating SQLE, and enhance SQLE protein stability through inhibition of the PI3K/Akt/GSK3β‐mediated proteasomal pathway. This establishes a synergistic relationship between SQLE and PTEN/p53 deficiencies, increasing cholesterol biosynthesis and promoting tumor cell growth and survival [138]. SQLE expression is specifically increased in HCC and is closely associated with poor clinical outcomes. SQLE significantly enhances HCC growth, epithelial–mesenchymal transition (EMT), and metastasis in both in vitro and in vivo models, with its effects on HCC being linked to the activation of the STRAP‐dependent TGF‐β/SMAD signaling pathway [139]. Furthermore, SQLE enhances the NADP^+^/NADPH ratio, which induces the expression of DNA methyltransferase 3A (DNMT3A), leading to the epigenetic silencing of phosphatase and tensin homolog (PTEN) and activation of oncogenic targets in the rapamycin signaling pathway [174]. In head and neck squamous cell carcinoma (HNSCC), inhibiting SQLE through a cholesterol‐dependent mechanism decreases the affinity of Akt for lipid rafts, leading to Akt inactivation at the lipid raft level, reducing GSK‐3β phosphorylation at S9, and increasing c‐Myc phosphorylation at T58, ultimately destabilizing c‐Myc and promoting HNSCC progression [140]. In esophageal squamous cell carcinoma (ESCC), LPCAT1 regulates the upregulation of the cholesterol synthesis enzyme SQLE by promoting PI3K activation. LPCAT1 also enhances the activation of HER (epidermal growth factor receptor) and PI3K, enabling SREBP1 to enter the nucleus with SP1/SREBPF2, thus promoting cholesterol synthesis [109]. Given the critical role of SQLE in tumorigenesis and progression, studying its expression in cancer and assessing its significance in patient prognosis is essential.

### 3.6 DHCR24

DHCR24 is involved in various cellular functions, including oxidative stress response, cellular differentiation, anti‐apoptotic activity, and anti‐inflammatory functions [42]. DHCR24 expression is elevated in BC compared to normal breast tissue, particularly in luminal and HER2‐positive BC specimens. Overexpression of DHCR24 enhances the population of BC stem‐like cells and increases the number of acetaldehyde dehydrogenase‐positive cells. Additionally, DHCR24 promotes the growth of cancer stem‐like cells by enhancing the Hedgehog signaling pathway [176]. DHCR24 is a direct target of the stem cell regulator SOX9, and in a diffuse large B‐cell lymphoma (DLBCL) cell line xenograft model, knockdown of SOX9 resulted in reduced DHCR24 levels, decreased cholesterol content, and reduced tumor load. These findings suggest that SOX9 drives lymphoma progression through the DHCR24 and cholesterol biosynthesis pathways, and the SOX9‐DHCR24‐cholesterol biosynthesis axis could serve as a novel therapeutic target for DLBCLs [141]. Given the potential therapeutic benefits of regulating serine/arginine‐rich splicing factor 3 (SRSF3) in CRC, silencing SRSF3 has been shown to significantly suppress CRC cell proliferation and migration by inhibiting its target gene, DHCR24. The novel SRSF3 inhibitor, SFI003, demonstrated strong antitumor efficacy both in vitro and in vivo, inducing apoptosis in CRC cells via the SRSF3/DHCR24/ROS pathway [142]. SH42, an inhibitor of DHCR24, reduces liver inflammation by preventing the activation of Kupffer cells and the infiltration of monocytes. However, the lack of LXRα completely abolishes these effects of SH42. Therefore, the inhibition of DHCR24 by SH42 prevents diet‐induced hepatic steatosis and inflammation in an LXRα‐dependent manner, without causing hyperlipidemia [143]. Chronic hepatitis C virus (HCV) infection triggers a transformation in liver cells that can lead to cancer. The increase in DHCR24 protein levels, prompted by the virus, protects against oxidative damage and cell death. This occurs concurrently with diminished acetylation of the critical protein p53 at specific sites within the nucleus, which usually plays a role in managing cellular stress responses. The data suggest that in the presence of hepatitis C, DHCR24 impairs the activation of p53 by promoting its association with MDM2, a molecule that regulates p53 levels in the cell, thus preventing p53 from executing its protective functions under cellular stress [144]. Therefore, DHCR24 could be an important target for HCV‐associated HCC therapy.

### 3.7 NPC1L1

NPC1L1 is the primary protein responsible for exogenous cholesterol absorption. R. J. Kwon et al. [177] found that NPC1L1 is highly expressed in CRC and is significantly correlated with N stage and pathological staging, suggesting that NPC1L1 could serve as an independent prognostic marker for CRC. R. Zhang et al. [178] analyzed “The role of NPC1L1 in cancer” and concluded that NPC1L1 knockdown significantly reduces the levels of inflammatory markers such as pc‐Jun, p‐ERK, and caspase‐1 p20 in CRC. Beyond CRC, NPC1L1 has also been studied in HCC. Kui Wang et al. evaluated the expression levels of NPC1L1 and NPC2 in HCC using immunohistochemistry, Western blot, and real‐time PCR and found that the expression of NPC1L1 and NPC2 was significantly lower in cancerous tissue compared to adjacent non‐cancerous tissue. The low expression of NPC1L1 and NPC2 in HCC tissues may indicate a poor prognosis for HCC patients [179]. Ezetimibe, an NPC1L1 inhibitor, impedes the PI3K/Akt/mTOR signaling pathway, inducing HCC. However, Ezetimibe effectively inhibits HCC cell proliferation via a non‐apoptotic cell death pathway—paraptosis [145]. These findings appear contradictory, and a reliable conclusion on whether NPC1L1 can serve as a therapeutic target for HCC is still lacking. The role of NPC1L1 in pancreatic ductal adenocarcinoma (PDAC) has also been widely researched. F. Guillaumond et al. [180] found through transcriptomic analysis that pancreatic cancer cells are highly dependent on cholesterol uptake. In 2017, through a multi‐omics analysis of 29 PDAC xenografts, the authors found that NPC1L1 inhibitors, such as Ezetimibe, are effective therapies for pancreatic cancer [181]. R. Zhang et al. [182] developed a potent dual inhibitor targeting pancreatic triglyceride lipase (PTL) and NPC1L1, P1‐11, which effectively reduced cholesterol absorption and demonstrated strong therapeutic effects on hypercholesterolemia [182]. Furthermore, in 2024, using transcriptome sequencing and data from the TCGA, ICGC, and GEO databases, the authors identified ADH1C, APOE, RAP1GAP, NPC1L1, P4HB, SOD2, and TNFSF10 as potential prognostic markers for PDAC [183]. These studies confirm that NPC1L1 can serve as a therapeutic target for pancreatic cancer. In addition to the cancers mentioned, it is noteworthy that in ovarian cancer, J. Yarmolinsky et al. [126] performed a genome‐wide association study (GWAS) meta‐analysis and found that in breast invasive carcinoma (BRCA) 1/2 gene mutation carriers, HMG‐CoA reductase inhibition was associated with a lower risk of ovarian cancer, while NPC1L1 and circulating LDL cholesterol inhibition were not significantly associated with epithelial ovarian cancer.

### 3.8 PCSK9

Proprotein convertase subtilisin/kexin type 9 (PCSK9) is a positive regulator of circulating LDL and cholesterol [184]. The liver is its primary site of expression [185]. The human PCSK9 gene is located on chromosome 1p32.3, spanning 22 kb, and consists of 12 exons and 11 introns. PCSK9 is synthesized in the ER as a 692‐amino acid precursor, which includes a C‐terminal cysteine/histidine‐rich domain (amino acids 453–692), a hinge region (amino acids 405–452), a catalytic domain (amino acids 153–404), a pro‐domain (amino acids 31–152), and an N‐terminal signal peptide (amino acids 1–30) [186]. The PCSK9 promoter contains binding sites for the transcription factors SREBF2 [69], HNF1α [187], E2F1 [188], and HINFP [189], all of which increase PCSK9 expression, while FOXO3 inhibits it [190]. Studies have shown that PCSK9 regulates signaling pathways such as PI3K/Akt [146], MAPK [191], and Wnt/β‐catenin [147], thus influencing tumor cell proliferation, survival, and angiogenesis. Pan‐cancer analysis of TCGA datasets revealed that PCSK9 is highly expressed in several cancers, including BRCA, cervical squamous cell carcinoma, colon adenocarcinoma, esophageal carcinoma, HNSCC, HCC, rectal adenocarcinoma, gastric adenocarcinoma, thyroid carcinoma, and endometrial carcinoma, and is significantly associated with advanced disease staging and poor prognosis [192]. In CRC, L. Wang et al. [146] analyzed clinical colon cancer tissues and found that PCSK9 is highly expressed, promoting tumor cell proliferation, migration, and invasion by regulating tumor cell EMT and PI3K/Akt signaling. Inhibition of PCSK9 effectively suppressed CRC development [146]. Similarly, C. C. Wong et al. [147] used CRC patient cohort analyses, homologous cell lines, and transgenic mice to discover that APC/KRAS mutant CRC induces de novo cholesterol synthesis, accompanied by an increase in geranylgeranyl pyrophosphate (GGPP), a metabolite required for KRAS activation, which promotes the development of APC/KRAS mutant CRC through activation of the KRAS/MEK/ERK signaling pathway. In contrast, inhibition of PCSK9 significantly suppressed APC/KRAS mutant CRC cell growth both in vitro and in vivo [147]. In 2023, M. Rada et al. [148] used clinically approved PCSK9‐neutralizing antibodies (Evolocumab) to inhibit PCSK9 in CRC liver metastases (CRLC), which led to the downregulation of runt‐related transcription factor‐1 (RUNX1) expression in CRLC cells. RUNX1 is positively correlated with vessel co‐option formation, and the authors suggested that PCSK9 inhibition is an effective therapeutic strategy for enhancing anti‐angiogenesis treatment. In HCC, S.‐Z. Zhang et al. [149] analyzed 105 clinical HCC samples and found that high PCSK9 expression in tumor tissues was associated with microvascular invasion and large tumor volume. Patients with high PCSK9 expression exhibited lower overall survival and disease‐free survival. Mechanistically, PCSK9 increases FASN expression, suppressing HCC cell apoptosis via the Bax/Bcl‐2/Caspase9/Caspase3 pathway, thus promoting HCC growth [149]. However, in April of the same year, M. He et al. [150] found that PCSK9 expression in HCC tissues was significantly lower than in adjacent non‐cancerous tissues. Clinical specimens revealed that patients with low PCSK9 expression had significantly shorter overall survival and disease‐free survival compared to those with high PCSK9 expression. Mechanistically, PCSK9 interacts with GSTP1, promoting the dissociation of GSTP1 dimers and inactivating the JNK signaling pathway in HCC cells, inhibiting HCC cell proliferation and metastasis [150]. These contradictory findings require further data to clarify the relationship between PCSK9 and HCC. Y. Sun et al. [151] discovered that PCSK9 overexpression induced AKT‐S473 phosphorylation, leading to HCC cell proliferation and sorafenib resistance. ZDHHC16 mediated PCSK9‐C600 (cysteine 600) S‐palmitoylation, which significantly increased the affinity between PCSK9 and PTEN, leading to PTEN degradation and subsequent AKT activation. The researchers created a bioactive peptide derived from PCSK9 that acts as a competitive inhibitor of PCSK9 palmitoylation, thereby reducing AKT phosphorylation and boosting the anti‐tumor efficacy of sorafenib in HCC [151]. They evaluated the effects of PCSK9 inhibition in HCC cell lines (Huh6, Huh7, HepG2) and verified the results in a zebrafish model. PCSK9 inhibition disrupted the p62/Keap1/Nrf2 antioxidant axis, effectively disrupting tumor metabolism, inducing metabolic exhaustion, and increasing cancer cell vulnerability to iron‐induced lipid peroxidation, thus suggesting that anti‐PCSK9 therapy is an effective treatment for liver cancer [152]. RNA sequencing and cell thermal transfer assays indicated that Flubendazole, by reducing PCSK9 expression, inhibited PCSK9‐induced Hedgehog signaling, leading to downregulation of Smoothened (SMO) and Gli1 in HCC. Flubendazole significantly inhibited HCC cell growth both in vitro and in vivo, and in combination with lenvatinib, it enhanced the anticancer effects of lenvatinib [153]. In 2024, using HepG2 cells for subcutaneous transplantation and luci‐Hepa1‐6 cells for orthotopic liver injection, the study found that high PCSK9 expression was negatively correlated with overall survival and CD8^+^ T cell markers in HCC patients. Mechanistically, PCSK9 promoted mTORC1 activation via LDLR in CD8^+^ T cells, and inhibiting PCSK9 significantly enhanced the efficacy of TCR‐T cell therapy and anti‐PD‐1 immunotherapy [154]. In conclusion, PCSK9 inhibition can effectively suppress HCC progression, regardless of whether PCSK9 expression is high or low. In addition to its roles in CRC and HCC, PCSK9 has also been shown to promote gastric cancer [155], BC [156], prostate cancer [157, 158], and non‐melanoma skin cancer (NMSC) [159], demonstrating its significant promoting role in these cancers (Table 1).

In conclusion, several enzymes in the cholesterol biosynthesis pathway play a promotive role in cancer development, particularly SREBPs, HMGCR, and PCSK9, which are closely associated with the progression of HCC, CRC, and bc. However, most current studies have primarily observed these phenomena, with limited research into the underlying mechanisms. Approved NPC1L1 inhibitors, such as Ezetimibe, and HMGCR inhibitors like statins, can be used in combination with anticancer drugs for cancer treatment. Similarly, PCSK9 inhibitors such as evolocumab and alirocumab can synergize with anti‐PD‐1 therapies to inhibit cancer growth. Despite these findings, these inhibitors have not yet been clinically applied to cancer treatment. Therefore, targeting the cholesterol biosynthesis pathway for cancer therapy remains an area requiring further development.

In recent years, the concept of the tumor microenvironment has significantly enhanced our understanding of tumor progression. Within the tumor microenvironment, two major mechanisms promote tumor development: cellular metabolic reprogramming [193] and immune evasion [194]. Therefore, the following discussion will focus on how abnormal cholesterol levels in the tumor microenvironment regulate tumor immunity.

## 4 Interaction Between Cholesterol Metabolism and Tumor Immune Microenvironment

An increasing body of evidence underscores the complex interactions between energy metabolism and immune cell responses [195]. Furthermore, the emerging field of immunometabolism seeks to elucidate the bidirectional causal relationship between metabolic reprogramming and immune dysfunction. Cancer itself undergoes metabolic reprogramming, which significantly disrupts the functions of immune cells within the tumor microenvironment [196]. The tumor microenvironment harbors a variety of tumor‐infiltrating immune cells, including cytotoxic T cells, B lymphocytes, tumor‐associated macrophages (TAMs), neutrophils, dendritic cells (DCs), regulatory T cells, myeloid‐derived suppressor cells (MDSCs), and natural killer cells [197].

### 4.1 Cytotoxic T Cells

An increasing body of evidence highlights the complex interactions between energy metabolism and immune cell responses [195]. Furthermore, the emerging field of immunometabolism seeks to elucidate the bidirectional causal relationship between metabolic reprogramming and immune dysfunction. Cancer itself undergoes metabolic reprogramming, which severely disrupts immune cell functions within the tumor microenvironment [196]. The tumor microenvironment contains various tumor‐infiltrating immune cells, including cytotoxic T cells, B lymphocytes, TAMs, neutrophils, DCs, regulatory T cells, MDSCs, and natural killer cells [197]. In tumor‐infiltrating CD8^+^ T cells, cholesterol content in tumor tissues is positively correlated with the upregulation of immune checkpoint markers such as PD‐1, 2B4, TIM‐3, and LAG‐3. Cholesterol promotes the expression of these immune checkpoints by enhancing ER stress in CD8^+^ T cells. This process activates the ER stress sensor XBP1, which regulates the transcription of PD‐1 and 2B4. Furthermore, inhibition of XBP1 or reduction of cholesterol levels in CD8^+^ T cells effectively restores their anticancer activity [198]. Notably, SREBPs are essential for the metabolic reprogramming of CD8^+^ T cells when responding to growth signals. In the absence of SREBPs, these T cells lose their ability to combat stromal cell components, leading to reduced cell proliferation under laboratory conditions and diminished clonal expansion during viral attacks [199]. In the tumor microenvironment, oxysterols promote reciprocal regulates between the LXR and SREBP2 pathways, resulting in cholesterol depletion in T cells. This depletion causes metabolic abnormalities that drive T cell exhaustion and dysfunction [200, 201]. In B16F10 melanoma and MC38 CRC models, cholesterol depletion was observed in T cells within the tumor microenvironment, while myeloid‐derived immunosuppressive cells and tumor cells exhibited cholesterol accumulation. Cholesterol deficiency induces autophagy‐mediated apoptosis in T cells. Oxysterols in the tumor microenvironment induce cholesterol depletion in T cells via modulation of SREBP2/LXR, with CD8^+^ T cells being more susceptible to cholesterol deficiency than CD4^+^ T cells [200]. Notably, cholesterol depletion in tumors inhibits mTORC1 signaling, leading to T cell exhaustion. In contrast, increasing cholesterol levels in chimeric antigen receptor (CAR)‐T cells by blocking LXR improves anticancer function [202]. FGF21 maintains the hyperactivation of the AKT–mTORC1‐SREBP1 signaling axis in activated CD8^+^ T cells, resulting in increased cholesterol biosynthesis and T cell exhaustion [203]. Youjun Li et al. found that increased cholesterol in the tumor microenvironment (TME) suppresses the cytotoxicity of CD4^+^ T cells. ZDHHC3 mediates S‐acylation of SCAP at C264, antagonizing HACE1‐mediated SCAP ubiquitination. This S‐acylation reprograms cholesterol metabolism and promotes HCC immune escape [204]. TIP analysis showed that the activity of “T cell recruitment” and “immune cell infiltration into tumors” was negatively correlated with HMGCR expression [129]. Researchers analyzed data from about 3.8 million cancer patients and 1.3 million LDL measurement participants from the UK Biobank and FinnGen and combined this with in vivo and in vitro experiments. They found that the combination of Lovastatin and paclitaxel enhances CD8^+^ T cell activity, improving BC prognosis [124]. In HCC, specific SQLE knockout inhibits tumor growth, increases cytotoxic CD8^+^ T cells, and decreases Arg‐1^+^ MDSCs, indicating that SQLE promotes immune suppression in MASH‐HCC [205].

Several studies indicate that PCSK9 is associated with the initiation of cancer progression [147, 206, 207]. Gene knockout or PCSK9 antibody inhibition increases the expression of MHC‐I proteins on the tumor cell surface, promoting strong infiltration of cytotoxic T cells within tumors and enhancing the efficacy of anti‐PD1 therapies [208]. Using HepG2 cells for subcutaneous transplantation and luci‐Hepa1‐6 cells for orthotopic liver injection, researchers found that high PCSK9 expression negatively correlated with overall survival and CD8^+^ T cell markers in HCC patients. The mechanism involves PCSK9 promoting mTORC1 activation via LDLR in CD8^+^ T cells, and inhibiting PCSK9 significantly enhances the efficacy of TCR‐T cell therapy and anti‐PD‐1 immunotherapy [154]. In CRC, Fumarase (FH) downregulation in tumors is associated with poor prognosis in CRC patients. In Fh1 knockout tumor mouse models, the combination of PCSK9 inhibitors and PD‐1 antibodies significantly improved the therapeutic effect of PD‐1. Mechanistically, FH interacts with Ras‐related nuclear protein (RAN), which inhibits the nuclear import of the PCSK9 transcription factors SREBF1/2, thereby reducing PCSK9 expression. This leads to increased CD8^+^ T cell clonal expansion, while Treg cell numbers remain unchanged and PD‐L1 expression does not significantly change, enhancing the immune response [209]. Additionally, researchers developed CaCO_3_ nanoparticles coated with immune cell death inducers DOX and evolocumab. The DOX/evolocumab‐loaded CaCO_3_ nanoparticles (DECP) showed good acid‐neutralizing capabilities, inducing immunogenic cell death in cancer cells. DECP, by targeting PCSK9, inhibited PCSK9 activity, increasing the proportion of mature DCs, tumor‐infiltrating CD8^+^ T cells, and natural killer cells in HCC, while depleting Foxp3^+^ regulatory T cells [210]. Yongjun Liu et al. developed pH‐responsive lipid nanoparticles (NL/PLDs) loaded with PCSK9 shRNA, lonidamine (LND), and low‐dose doxorubicin (DOX), which effectively alleviated the tumor immune‐suppressive microenvironment (TIME) and enhanced CD8^+^ T cell‐mediated anticancer immunity. Mechanistically, NL/PLDs enhance tumor immunogenicity, increase MHC‐I expression on tumor cells, and alleviate the inhibitory effect of tumor‐secreted lactate (LA) on T cell effector functions, sending activation, recognition, and killing signals, thereby awakening T cell function in the melanoma immune microenvironment in mice [211].

### 4.2 B Cells

SREBP pathway is essential for maintaining cholesterol balance during B cell activation and germinal center B cell proliferation. Lipopolysaccharides activate B cells through the Toll‐like receptor 4 (TLR4)‐related signaling pathway, which in turn activates the nuclear factor κ‐light chain enhancer. This activation increases the expression of SREBPs, thereby enhancing lipid synthesis and storage [212]. Bali Pulendran and colleagues created mouse models with specific SCAP knockout in B cells or CD11c^+^ antigen‐presenting cells (APCs), where SCAP is a crucial regulator of the SREBP signaling pathway. Their findings revealed that the loss of SCAP in CD11c^+^ APCs did not notably affect immune responses. However, the SREBP pathway played a key role in the metabolic reprogramming of activated B cells. In SCAP‐deficient B cells, mitogen‐induced proliferation was impaired, and lipid raft levels were reduced. Additionally, SCAP knockout in germinal center B cells via AID‐Cre led to a decrease in lipid raft content and disrupted cell cycle progression [213]. In human B cell lymphoma samples, SREBP2 protein is highly expressed, and B cell lymphoma cells respond to statin therapy by activating the mTORC1‐pS6 pathway. Therefore, combining low‐dose statins with the mTOR inhibitor rapamycin produces a synergistic effect, significantly inhibiting B cell lymphoma proliferation, cell cycle progression, and lipid raft formation [119].

### 4.3 Tumor‐Associated Neutrophils

Cancer‐derived cholesterol metabolites, particularly oxysterols, have varying effects on the function of different tumor‐infiltrating immune cells. Tumor‐derived oxysterols can bind to and activate the G protein‐coupled receptor C‐X‐C motif chemokine receptor 2 (CXCR2), rather than the LXR, to recruit neutrophils, promote angiogenesis, and induce immunosuppression, thereby facilitating tumor growth [214]. Additionally, in pancreatic neuroendocrine tumors, the increase in 24S‐hydroxycholesterol, induced by hypoxia‐inducible factor‐1α (HIF1α), attracts neutrophils to the tumor microenvironment, promoting angiogenesis [215]. In a BC model on a high‐cholesterol diet, 27HC was observed to recruit polymorphonuclear neutrophils and γδ T cells, while reducing the presence of cytotoxic CD8^+^ T cells, ultimately facilitating tumor metastasis [216].

### 4.4 Tumor‐Associated Macrophages

Macrophages inherently possess the ability to suppress tumors, but in the tumor microenvironment, due to the metabolic reprogramming of tumor cells, macrophages are influenced and undergo a phenotypic transformation. These TAMs support tumor growth through immune suppression and facilitation. Toby Lawrence et al. characterized TAMs in a metastatic ovarian cancer mouse model and found that ovarian cancer cells secrete hyaluronic acid oligomers, which lead to cholesterol efflux and lipid raft depletion in macrophages. This process promotes IL‐4‐mediated reprogramming, including the inhibition of IFNγ‐induced gene expression, and drives TAMs toward a pro‐tumor M2‐like phenotype. Knockout of ABC transporters mediating cholesterol efflux reversed the pro‐tumor functions of TAMs and reduced tumor progression [217]. In macrophages, 25HC induces cytoskeletal rearrangement to attract macrophages, possibly by upregulating matrix metalloproteinases (MMPs), thus promoting cancer metastasis [218]. The nuclear receptor subfamily 4 group A member 2 (NR4A2) activates SQLE, leading to the dysregulation of microglial cholesterol homeostasis. This, through the NR4A2‐SQLE axis, enhances oxidative stress and promotes tumor growth. Targeting SQLE can enhance the therapeutic effects of immune checkpoint blockade [219]. Squalene epoxidase (SQLE) is required for β‐glucan‐induced macrophage training immunity and subsequent anticancer activity. 24(S),25‐epoxycholesterol (24(S),25‐EC), a metabolite of the cholesterol biosynthesis branch, activates the LXR and increases chromatin accessibility, thereby invoking innate immune memory. Meanwhile, SQLE‐induced ROS accumulation stabilizes hypoxia‐inducible factor 1α (HIF‐1α) protein, leading to a metabolic shift to glycolysis [220]. Jiwu Wei et al. found that in male mouse GBM models, excessive cholesterol content in tumors induces dysfunction in monocyte‐derived TAMs, leading to disease progression. Further analysis of cholesterol metabolism in TAMs sorted in vivo revealed that apolipoprotein A1 mediates lipid‐related metabolic remodeling and decreases 7‐ketocholesterol levels, directly inhibiting TNF signaling in TAMs by suppressing mitochondrial translation [221]. Through metabolomics, researchers found that 7α,25‐dihydroxycholesterol (7α,25‐OHC) concentrations are elevated in the plasma of patients with systemic lupus erythematosus. 7α,25‐OHC, upon binding to the receptor Epstein–Barr virus‐induced gene 2 (EBI2) in macrophages, inhibits STAT activation and the production of IFN‐β, chemokines, and cytokines. The expression of EBI2 is significantly reduced in monocytes/macrophages of systemic lupus erythematosus patients and mice, while IFN‐γ produced by activated T cells triggers EBI2 expression. Macrophages lacking EBI2 produce higher levels of chemokines and cytokines, thereby recruiting and activating myeloid cells, T lymphocytes, and B lymphocytes, which exacerbates thioacetamide‐induced systemic lupus erythematosus [222].

PCSK9 also plays a significant role in macrophage polarization. Shuwen Yu et al. analyzed clinical colon cancer tissues and found that PCSK9 is highly expressed in colon cancer. PCSK9 knockout inhibits M2 macrophage polarization but also promotes M1 macrophage polarization by reducing LA, protein emulsion, and macrophage migration inhibitory factor (MIF) levels [146]. Researchers established Hepa1‐6 liver cancer mouse xenografts and a co‐culture system of Hepa1‐6 cells and mouse primary macrophages to evaluate the effects of Arenobufagin (ARBU) on tumor progression and macrophage polarization. They found that ARBU inhibited cholesterol synthesis in the tumor microenvironment via the PCSK9/LDL‐R signaling pathway, blocking M2 macrophage polarization, promoting tumor cell apoptosis, and inhibiting tumor cell proliferation and migration [223].

### 4.5 Myeloid Dendritic Cells

DCs play a crucial role in the initiation and regulation of immune responses. Alterations in DC function can lead to immune dysregulation. In DCs, specific oxysterols bind to LXRα, leading to the downregulation of CCR7, thereby impairing their function [224]. In tumor‐bearing mice (tumor cell lines: RMA, B16, and TrampC1), blocking cholesterol synthesis or inactivating the LXR‐α ligand by expressing SULT2B1b (a cholesterol sulfotransferase that converts oxysterols to inactive sulfated oxysterols) can restore DC function and enhance anticancer responses [225]. ABCA1/G1 knockout in DCs prevents cholesterol efflux, resulting in an increase in CD11b^+^ DCs in the lymph nodes and spleens, leading to symptoms of systemic lupus erythematosus (lymphadenopathy and glomerulonephritis). Concurrently, granulocyte‐macrophage colony‐stimulating factor receptor levels on the cell surface increase, and there is enhanced secretion of inflammatory cytokines [226]. A protein secreted by HCC, α‐fetoprotein (AFP), reduces the expression of SREBP1 and PGC1‐α in DCs, resulting in decreased fatty acid synthesis and mitochondrial metabolism [227]. Vincenzo Russo et al. disrupted oxysterol metabolism from both genetic and pharmacological perspectives, analyzing the distribution of tumor lipid bodies associated with tumor‐infiltrating immune cells. They found that sulfotransferase 2B1b, which is crucial for cholesterol and sulfated oxysterol synthesis, disrupts lipid bodies in the tumor microenvironment, facilitating the infiltration of monocyte‐derived antigen‐presenting cells (including monocyte‐DCs) into the tumor. Treatment with oxysterol receptor LXR antagonists in Lewis lung cancer mice promoted monocyte‐DC differentiation within the tumor, slowed tumor growth, and synergized with anti‐PD‐1 immunotherapy and CAR‐T cell therapy [228]. Through in vitro co‐culture and tumor mouse models, the immune‐modulatory effects of PCSK9 inhibition were evaluated. PCSK9 inhibition was found to upregulate DC infiltration and MHC‐II expression, improving the activation of CD8^+^ T cells in the tumor immune microenvironment and effectively controlling tumor progression. The combination of PCSK9 inhibitors with OVA‐II tumor vaccines enhanced therapeutic efficacy [192].

### 4.6 Regulatory T Cells

Regulatory T cells (Tregs) suppress immune cell activation through cell contact or the secretion of inhibitory cytokines. In tumors, they assist cancer cells in evading immune responses. Dario A. A. Vignali et al. analyzed the intratumoral immune environment of Foxp3^Cre‐YFP^ or Nrp1^L/L^Foxp3^Cre‐YFP^ mice inoculated with B16.F10 melanoma (B16) or MC38 adenocarcinoma and found that Treg cells inhibit the secretion of interferon‐γ (IFNγ) by CD8^+^ T cells. IFNγ would otherwise block SREBP1‐mediated immune‐suppressive (M2‐like) TAM fatty acid synthesis. Therefore, Treg cells indirectly but selectively maintain the metabolic fitness, mitochondrial integrity, and survival of M2‐like TAMs [229]. Deletion of FASN in Treg cells inhibits tumor growth. Blocking PD‐1 or SREBP signaling leads to dysregulation of phosphoinositide 3‐kinase (PI3K) activation in intratumoral Treg cells [230]. In a study using AMPKα^1fl/fl^Foxp3^YFP‐Cre^, Foxp3^YFP‐Cre^, Rag1^−/−^, and C57BL/6 J mice, researchers found that the absence of the AMPKα1 subunit in Tregs enhances glycolysis and increases the expression of HMGCR. Furthermore, AMPK activates p38 mitogen‐activated protein kinase (MAPK), phosphorylates glycogen synthase kinase‐3β (GSK‐3β), and suppresses PD‐1 expression in Tregs [231].

### 4.7 Myeloid‐Derived Suppressor Cells

MDSCs are immature myeloid cells derived from hematopoietic stem cells. While present in low numbers in healthy individuals, they proliferate extensively in inflammatory and tumor environments. MDSCs exert immunosuppressive functions through various pathways and mechanisms, thereby indirectly suppressing the host's immune response. In HCC derived from nonalcoholic steatohepatitis (NASH), androgen receptor (AR)‐driven oncogene cell cycle‐related kinase (CCRK) induces STAT3‐AR promoter co‐occupancy and transcriptional upregulation, leading to GSK3β phosphorylation, activation of the mTORC1/4E‐BP1/S6K/SREBP1 cascade, and enhanced recruitment and tumorigenicity of MDSCs [232]. In the tumor microenvironment, the function of the ER in both tumor cells and immune cells is disrupted, triggering a defense mechanism known as the unfolded protein response (UPR). This sustained response helps tumors evade immune surveillance. Importantly, during this process, X‐box binding protein 1 (XBP1) enhances cholesterol generation and release, stimulating MDSCs, which leads to immune system suppression. These MDSCs receive cholesterol encapsulated in small vesicles and ingest it through a process similar to macrophage phagocytosis of particles. Genetic or pharmacological depletion of XBP1 significantly reduces the abundance of MDSCs and triggers a strong antitumor response when tumor cholesterol levels are reduced [233]. Many mechanisms remain unexplored regarding how MDSCs promote tumor growth, warranting further investigation.

**FIGURE 4:**
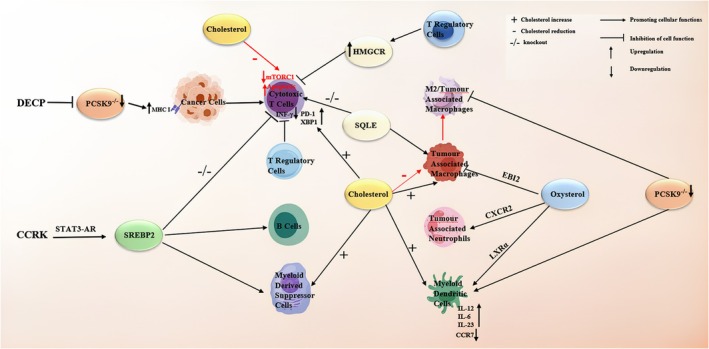
The interaction between cholesterol metabolism and tumor‐infiltrating immune cells.

The balance of cholesterol metabolism is crucial for maintaining normal immune cell function. However, in the tumor microenvironment, the reprogramming of cholesterol metabolism by tumor cells leads to an imbalance in cholesterol homeostasis. Specifically, immune cells within the tumor microenvironment often experience cholesterol deficiency, resulting in immune cell dysfunction. The underlying mechanisms of cholesterol deficiency in immune cells in the tumor microenvironment; however, still require further in‐depth investigation (Figure 4).

## 5 Targeting Cholesterol to Treat Diseases

### 5.1 LXR

The LXR agonist RGX‐104 effectively inhibits the growth of various mouse and human tumors. By upregulating the LXR transcriptional target APOE, RGX‐104 depletes MDSCs, which in turn enhance T cell activation. Notably, this finding was further confirmed in cancer patients during a phase I clinical trial. Additionally, LXR activation has been shown to enhance other immunotherapies, including adoptive T‐cell transfer and checkpoint blockade therapy, in mouse models [234]. Additionally, RGX‐104 partially mitigated the immunosuppressive effects of radiotherapy in a mouse model of NSCLC [235].

### 5.2 SREBP

Adiponectin, a specific inhibitor of SREBP activation, is a diarylthiazole derivative that interacts with SCAP to prevent the translocation of SREBP1 and SREBP2 from the ER to the Golgi apparatus [236]. In prostate cancer, adiponectin inhibits cell proliferation and colony formation in both androgen‐responsive and androgen‐insensitive cancer cells, inducing G2/M cell cycle arrest and apoptosis. This effect is mediated by the inhibition of SREBP‐regulated metabolic pathways and androgen receptor (AR) signaling networks [237]. Furthermore, adiponectin can reverse progesterone resistance by inhibiting the SREBP1/NF‐kB pathway in endometrial cancer [238]. In addition to restraining SREBP activity, this agent also obstructs the formation of the mitotic spindle necessary for cell division in aggressive tumor types [239]. Furthermore, tocotrienol, a less common form of vitamin E, selectively breaks down mature SREBP2 without disturbing LXR functions, thus ensuring cholesterol balance in prostate cancer cells [240]. Similarly, rhodopsin, a compound extracted from certain plants, curtails the transcriptional prowess of SREBP‐2, interferes with cholesterol synthesis, and dampens the Akt pathways, thereby heightening the vulnerability of HCC cells to the tumor‐fighting properties of sorafenib in both lab cultures and animal studies [241].

A recently developed androgen receptor (AR) antagonist, psalutamide, significantly inhibited proliferation and migration, induced cysteine protease‐dependent apoptosis, and decreased lipid droplet levels in prostate cancer (PCa) cells by modulating the levels of ACL, ACC, FASN, and SREBP1. Furthermore, proxalutamide reduced AR expression in PCa cells, potentially overcoming resistance to AR‐targeted therapies [242].

### 5.3 HMGCR

Targeting HMGCR, a key enzyme in cholesterol synthesis, is considered one of the strategies for the treatment of cancer [243]. Initially developed for the treatment of cardiovascular diseases, statins have now become a standard therapeutic approach for cancer patients presenting with elevated cholesterol levels [244]. Statins competitively inhibit HMG‐CoA reductase in its controlled conversion of HMG‐CoA to mevalonate [245]. In the TME, statins can reduce tumor cell proliferation, promote apoptosis, induce autophagy, reduce migration and invasion, and promote anti‐inflammatory immunomodulation by affecting key proteins such as Ras, RhoA/C, Rac, and Rab [246]. In smooth muscle tumor experiments, simvastatin treatment was found to reduce cell proliferation, enhance apoptosis, and decrease extracellular matrix protein levels [247]. Additionally, synergistic effects were observed when statins were used alongside anti‐PD‐1 therapy [248].

### 5.4 FPPS

Bisphosphonates laced with nitrogen, serving as inhibitors of FPPS, mark a distinguished group of compounds aimed at the mevalonate pathway [249]. These N‐BPs bind more strongly to hydroxyapatite than their predecessors and effectively interrupt the FPPS function in this pathway. The newer, third‐generation N‐BPs like zoledronic acid and minodronate show superior inhibition of FPPS compared to earlier bisphosphonates. Their enhanced abilities extend to curbing cancer cell growth, promoting cell death, hindering the formation of new blood vessels, and lessening the likelihood of cancer cells clinging to the bone, showcasing their versatility against different cancer types through various actions [250]. Due to its strong inhibitory effect on osteoclasts, N‐BP is used for the treatment of osteolytic bone metastases, and it is also commonly used in the advanced treatment of prostate and BC [251].

### 5.5 SQLE

**FIGURE 5:**
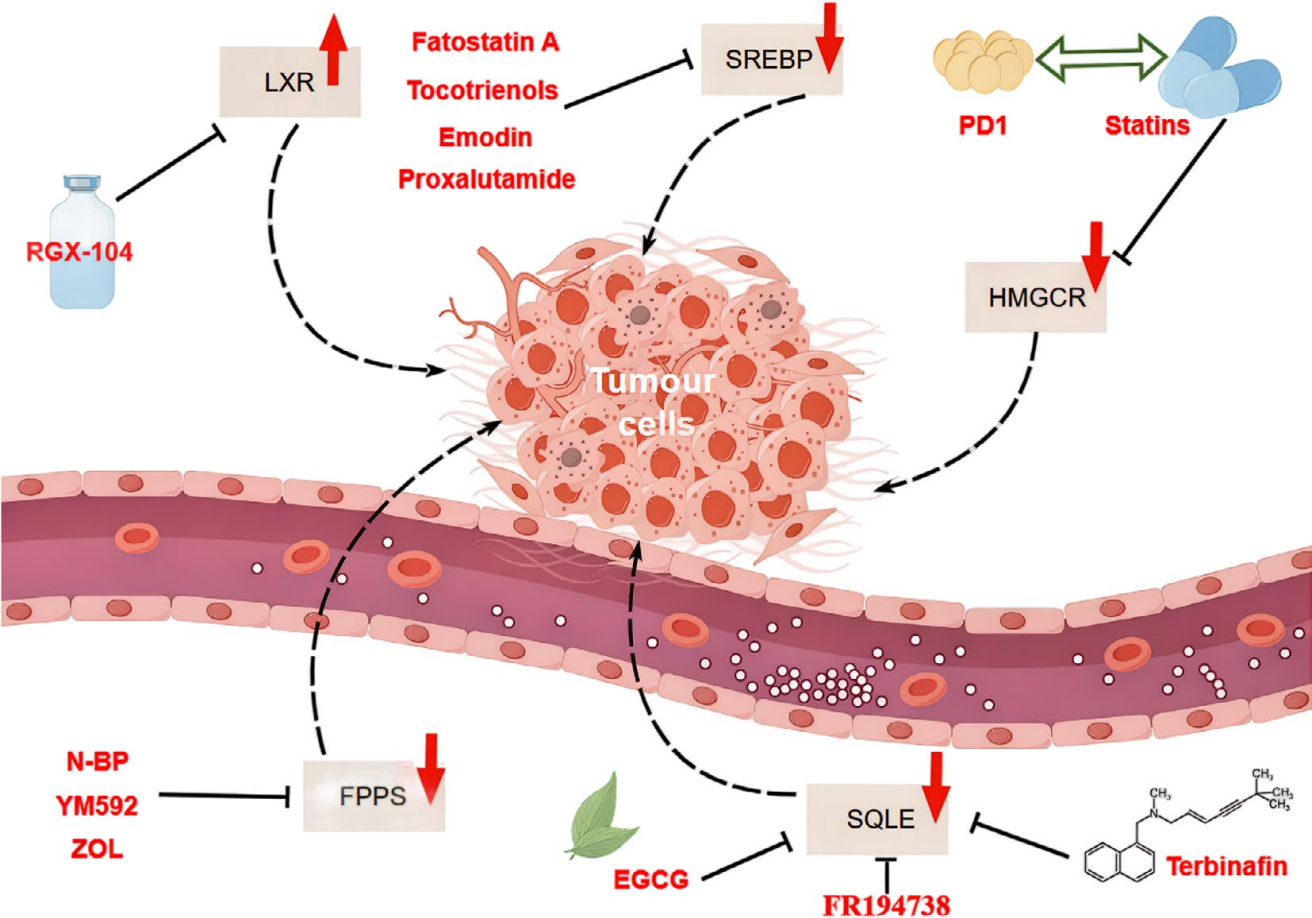
Targeting regulatory elements and key enzymes of cholesterol metabolism for cancer therapy.

Given the dysregulation of SQLE in cancer and its tumor‐promoting function, targeting SQLE is considered a new and promising antitumor therapy. Terbinafine, a pioneer SQLE inhibitor used in antitumor therapy (Figure 2), was shown to reduce the overall risk of death in a retrospective cohort study of prostate cancer patients receiving systemic administration of terbinafine [252], and another study showed that terbinafine reduced PSA levels in three‐quarters of patients with advanced prostate cancer [253]. In HCC induced by non‐alcoholic fatty liver disease (NAFLD), terbinafine promotes SQLE degradation via autophagy and subsequently restores PTEN expression, effectively inhibiting the AKT/mTOR signaling pathway [174]. Several SQLE inhibitors, encompassing natural substances and their modified forms, show potential as anti‐cancer agents or reliable inhibitors. Take Epigallocatechin 3‐O‐gallate (EGCG) from green tea, for example, which effectively curbs SQLE activity and maintains a commendable safety profile, even when administered in substantial quantities [254]. While the antitumor effects of EGCG have been widely investigated, the specific role of the SQLE‐EGCG interaction in mediating these effects remains unclear and warrants further detailed study [255]. Single‐cell RNA sequencing (scRNA‐seq) has revealed that the overexpression of SQLE in osteosarcoma (OSA) cells may serve as a potential therapeutic target. Furthermore, the SQLE‐specific inhibitor FR194738 has demonstrated anti‐OSA effects in vivo and exhibits synergistic interactions with chemotherapeutic agents [256] (Figure 5).

## 6 Conclusions

Cholesterol plays a crucial role in maintaining the structural and functional properties of the cell membrane bilayer. A substantial body of evidence indicates that elevated cholesterol levels are associated with the development of cancer. Cancer cells require a continuous supply of cholesterol to sustain abnormal proliferation, much of which is provided by de novo synthesis of acetyl coenzyme A in the ER. Cholesterol reprogramming in the tumor microenvironment supplies a significant amount of cholesterol to tumor cells, thereby promoting tumor progression. The reprogramming of cholesterol metabolism in tumors is driven by both endogenous and exogenous factors. Endogenous factors include the activation of oncogenes and the inactivation of tumor suppressor genes, while exogenous factors include ER stress, microenvironmental acidification, and inflammatory factors. Consistent with this, cholesterol reprogramming also affects immune cells in the tumor microenvironment and their immune functions, facilitating immune evasion by tumor cells. Numerous clinical and preclinical studies have shown that interfering with cholesterol metabolism in tumor cells and immune cells can be an effective strategy for tumor treatment. Cholesterol metabolism modulation can also be combined with existing clinical treatments to enhance the efficacy of cancer therapies. Further understanding of the role of cholesterol regulation, sterol regulatory element‐binding proteins, and enzymes involved in cholesterol‐regulated metabolism in cancer cells may provide effective targets for intervening in the biological functions of cancer cells and offer new opportunities for the design of novel cancer therapies.

Although significant progress has been made in cholesterol research, many important questions remain unanswered. For example, is it possible to identify a potent target within cholesterol biosynthesis pathways that can simultaneously combat tumor cell proliferation and metastasis while enhancing immune responses to eliminate tumor cells? What would be the optimal approach to effectively target cancer cells from multiple angles? Could drugs currently used for lipid metabolism disorders have new applications in cancer treatment? How can these drugs be effectively delivered to tumor cells? These questions urgently require further investigation.

## Author Contributions

**Yongmei Wu:** formal analysis, writing – review and editing, investigation. **Wenqian Song:** writing – original draft, investigation. **Min Su:** supervision, funding acquisition. **Jing He:** investigation, funding acquisition. **Rong Hu:** funding acquisition, supervision. **Youbo Zhao:** supervision, investigation, funding acquisition, visualization.

## Conflicts of Interest

The authors declare no conflicts of interest.

## Data Availability

The data that support the findings of this study are available from the corresponding author upon reasonable request.
